# Novel c-Jun N-Terminal Kinase (JNK) Inhibitors with an 11*H*-Indeno[1,2-*b*]quinoxalin-11-one Scaffold

**DOI:** 10.3390/molecules26185688

**Published:** 2021-09-20

**Authors:** Serhii A. Liakhov, Igor A. Schepetkin, Olexander S. Karpenko, Hanna I. Duma, Nadiia M. Haidarzhy, Liliya N. Kirpotina, Anastasia R. Kovrizhina, Andrei I. Khlebnikov, Irina Y. Bagryanskaya, Mark T. Quinn

**Affiliations:** 1 A.V. Bogatsky Physico-Chemical Institute, National Academy of Sciences of Ukraine, 65080 Odessa, Ukraine; sergey_a_lyakhov@ukr.net (S.A.L.); alex_chem_2@ukr.net (O.S.K.); dumaanna915@gmail.com (H.I.D.); 2 Department of Microbiology and Cell Biology, Montana State University, Bozeman, MT 59717, USA; igor@montana.edu (I.A.S.); liliya@montana.edu (L.N.K.); 3 Odessa National Polytechnic University, 65080 Odessa, Ukraine; nadezhdagajjdarzhi@gmail.com; 4 Kizhner Research Center, Tomsk Polytechnic University, 634050 Tomsk, Russia; anaskowry@gmail.com (A.R.K.); aikhl@chem.org.ru (A.I.K.); 5 Vorozhtsov Institute of Organic Chemistry, Siberian Branch, Russian Academy of Sciences, 630090 Novosibirsk, Russia; bagryan@nioch.nsc.ru

**Keywords:** c-Jun N-terminal kinase, kinase inhibitor, 11*H*-indeno[1,2-*b*]quinoxalin-11-one, oxime, interleukin-6, nuclear factor-κB

## Abstract

c-Jun N-terminal kinase (JNK) plays a central role in stress signaling pathways implicated in important pathological processes, including rheumatoid arthritis and ischemia-reperfusion injury. Therefore, inhibition of JNK is of interest for molecular targeted therapy to treat various diseases. We synthesized 13 derivatives of our reported JNK inhibitor 11*H*-indeno[1,2-*b*]quinoxalin-11-one oxime and evaluated their binding to the three JNK isoforms and their biological effects. Eight compounds exhibited submicromolar binding affinity for at least one JNK isoform. Most of these compounds also inhibited lipopolysaccharide (LPS)-induced nuclear factor-κB/activating protein 1 (NF-κB/AP-1) activation and interleukin-6 (IL-6) production in human monocytic THP1-Blue cells and human MonoMac-6 cells, respectively. Selected compounds (**4f** and **4m**) also inhibited LPS-induced c-Jun phosphorylation in MonoMac-6 cells, directly confirming JNK inhibition. We conclude that indenoquinoxaline-based oximes can serve as specific small-molecule modulators for mechanistic studies of JNKs, as well as potential leads for the development of anti-inflammatory drugs.

## 1. Introduction

c-Jun N-terminal kinase (JNK) is a member of the mitogen-activated protein kinase (MAPK) family, which are activated in response to various stress stimuli—such as ultraviolet radiation, oxidative stress, heat and osmotic shock, and ischemia-reperfusion injury of the brain and heart [1,2,3,4]. JNK has at least three isoforms with different functions, and identifying specific selective inhibitors is essential to the development of targeted therapeutics [5]. JNK1 and JNK2 are found in all cells and tissues of the body, whereas JNK3 is expressed mainly in the brain, heart, and testicles [2]. Previous studies show that JNKs play an important role in the regulation of signaling pathways involved in inflammation, apoptosis, and necrosis [6,7,8,9] and in a wide range of diseases, including multiple sclerosis, rheumatoid arthritis, osteoarthritis, insulin resistance, inflammatory bowel diseases, cancer, stroke, renal ischemia, essential hypertension, Alzheimer’s disease, and Parkinson’s disease [10,11,12,13,14,15,16,17,18,19,20,21,22,23].

**Figure 1:**
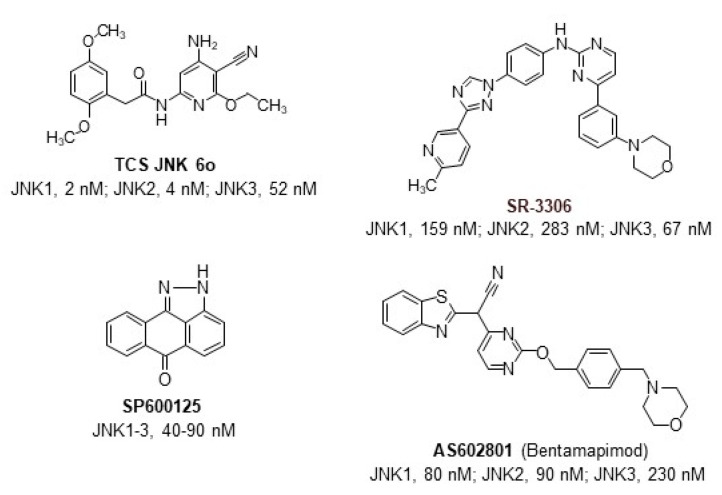
The reported scaffolds of JNK inhibitors and range of their inhibitory activity (IC_50_ values) [25,32,33,34].

Pharmacological and genetic evidence suggests that inhibition of JNK signaling may represent a promising therapeutic strategy [5,24]. To date, numerous medicinal chemistry efforts and high throughput screens have been initiated in efforts to identify selective and nontoxic JNK inhibitors with high therapeutic potential (Figure 1). For example, the JNK inhibitor AS602801 (Bentamapimod) has been shown to be effective and safe in clinical trials for the treatment of inflammatory endometriosis [25]. However, this compound inhibits all three JNK isoforms with similar efficacy [26]. Since JNK isoforms have been demonstrated to function independently and have distinct tissue expression patterns [17], the development of JNK isoform-specific inhibitors may have greater clinical benefit. Notably, JNK3 is expressed in the brain and the heart [26], so the development of selective inhibitors for this isoform may be promising in treating neurodegenerative diseases and other inflammatory disorders [27,28,29,30,31]. Furthermore, the therapeutic efficacy of lead compounds should be evaluated in appropriate ischemia/reperfusion models.

Previously, we identified a new class of JNK inhibitors based on the 11*H*-indeno[1,2-*b*]quinoxalin-11-one and indolo[2,1-*b*]quinazoline-6,12-dione scaffolds [35,36]. Specifically, compound **IQ-1** (11*H*-indeno[1,2-*b*]quinoxalin-11-one oxime) and its analogs inhibited JNK activity and, consequently, proinflammatory cytokine production by human myeloid cells [35,36,37,38,39]. These JNK inhibitors required the presence of an oxime group (Table 1), and compound C containing a Cl atom at position R^3^ had greater selectivity for JNK1/3 compared to JNK2 [36]. Based on these results, we suggested that modification of **IQ-1** could increase potency and/or a selectivity of the resulting analogs toward the three JNK isoforms.

In the present study, novel 11*H*-indeno[1,2-*b*]quinoxalin-11-one analogs with various substituents on the tetracyclic moiety (mainly at the R^3^ position or a combination of R^3^ with substituents at R^1^/R^2^) were synthesized and evaluated for their effects on JNK1-3. Compound design was based on introducing different types of substituents (i.e., strong and moderate electron donors/acceptors) into the quinoxaline moiety of **IQ-1** using correspondingly substituted 1,2-diaminobenzenes as the starting reagents. We also evaluated the anti-inflammatory potential of these compounds using in vitro cell-based assays.

## 2. Results and Discussion

### 2.1. Chemistry

**Scheme 1:**
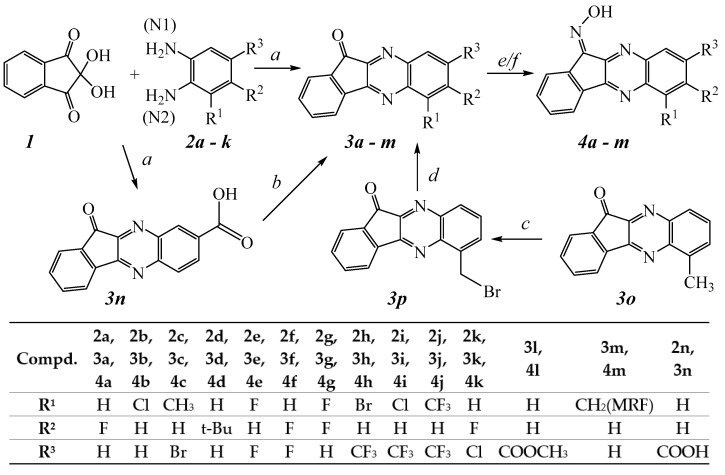
Reagents and conditions: (a) AcOH, 100 °C, 30 min, 35–90%; (b) CDI, MeONa, dimethylformamide, 25 °C, 1 h, 80%; (c) NBS, CCl_4_, 90 °C, 2 h, 80%; (d) R_2_NH; THF; 25 °C, 25–30%; (e) NH_2_OH∙HCl, C_5_H_5_N, EtOH, 100 °C, 4 h, 90–95%; (f) NH_2_OH∙HCl, NaOH, 80 °C, 2 h, 90–95%. Abbreviations: MRF—morpholin-1-yl.

All compounds were synthesized as shown in Scheme 1, and their structures were confirmed on the basis of analytical and spectral data. The simplest way to synthesize 11*H*-indeno[1,2-*b*]quinoxalin-11-one is the condensation of ninhydrin (**1**) with *o*-phenylenediamine [40,41]. Ketones **3a**–**3o** were synthesized via ninhydrin cyclocondensation with substituted 1,2-diaminobenzenes **2a**–**2k** in hot acetic acid, as described, and with comparable yields [42,43]. The reaction proceeded with the formation of a mixture of regioisomers; however, the content of the minor isomer did not exceed 10% in most cases. Ester **3l** was synthesized by esterification of previously synthesized **3n** [43] using 1,1′-carbonyldiimidazole and sodium methylate. Bromination of previously synthesized [43] 6-methyl-11*H*-indeno[1,2-*b*]quinoxalin-11-one (**3o**) led to the formation of 6-bromomethyl-11*H*-indeno[1,2-*b*]quinoxalin-11-one (**3p**). Aminodebromination of **3p** with morpholine, as described [44], led to the formation of **3m**.

Oximes **4a**–**4l** were synthesized from the corresponding ketones by condensation with hydroxylamine hydrochloride in a pyridine/ethanol mixture under heating due to the high solubility of **3a**-**3l** in pyridine. Oxime **4m** was synthesized by treating **3m** with a 5-fold excess of hydroxylamine hydrochloride in ethanol in the presence of NaOH in equimolar quantities.

Nucleophilic attack of the NH_2_ group is directed preferably to the C-2 atom of the ninhydrin molecule [45,46]. Thus, regioselective formation of ketones **3a-k** and **3n** (Scheme 1) is in general agreement with the charge distribution calculated for the precursor substituted 1,2-diaminobenzene molecules **2a-k**, **2n** using the DFT method with a B3LYP/aug-cc-pVDZ level of theory. The nitrogen atom Mulliken charges obtained for these diamines are shown in Table 2.

According to the Mulliken charge distribution, most of the precursor diamines should react with ninhydrin and form the ketones shown in Scheme 1, except for the difluorodiamines used in the synthesis of compounds **3e** and **3g**. In these cases, the observed regioselectivity results from the strong *ortho*-effect of the fluorine atom located next to one of the NH_2_ groups in the diamine (e.g., specific solvation of the fluorine atom by acetic acid used as the solvent). In diaminobenzene **2c**, the values of nitrogen charges are very close to each other. Thus, steric effects of the methyl substituent next to one of the NH_2_ groups leads to formation of ketone **3c** as the major regioisomer.

**Figure 2:**
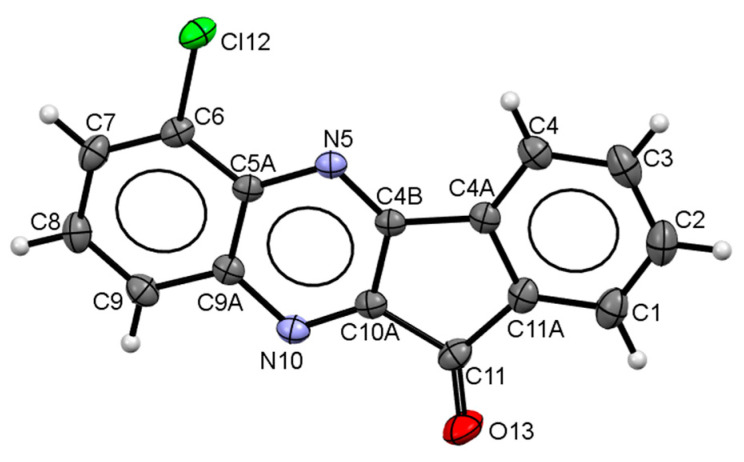
Molecular structure of compound **3b** determined by X-ray diffraction analysis.

The observed regioselectivity of ketone formation on the first stage (Scheme 1) was additionally confirmed for one of the synthesized ketones (**3b**) using single-crystal X-ray diffraction analysis. This compound formed crystals of high quality and size sufficient for this analysis. The crystal structure of **3b** is formed by two crystallographically-independent molecules. Molecular geometry of **3b** is shown in Figure 2 (one crystallographically-independent molecule is shown). The crystal structure was analyzed for short contacts between non-bonded atoms using the PLATON [47] and MERCURY programs [48]. The bond lengths and bond angles are very close for the two crystallographically-independent molecules and correspond to the statistical means [49]. According to the of X-ray diffraction data, molecules of **3b** are perfectly planar in the crystal.

The crystal structure of **3b** is composed of infinite π-stacks. Neighboring molecules are arranged in zero-inclined slipped-parallel fashion with head-to-head orientations. The separation of molecular planes within the 3.44 and 3.47 Å stacks are typical for π–stacking interactions [47]. The stacks are additionally linked by H-bonds of C-H…N and C-H…O types. These π-stacks and H-bond interactions lead to the formation of 3D networks in the crystal packing of **3b** (see Supplementary Materials Table S1 and Figure S1).

### 2.2. Binding Affinity of Novel Compounds toward JNK1-3

All compounds were evaluated for their ability to bind to JNK1-3 and compared with previously reported compound **IQ-1 [35]**. Four compounds (**4a, 4b, 4e,** and **4l**) had high affinity for JNK, with K_d_ values in the submicromolar range for all three JNK isoforms, whereas **4f** was more selective and had high affinity for JNK1 and JNK3 but did not bind to JNK2 (Table 3). Introduction of *t*-Bu at R^2^ led to compound **4d**, which had relatively low affinity for JNK. On the other hand, introduction of a fluoride atom as R^2^ or a chlorine substituent at R^1^ led to compounds **4a** and **4b**, respectively, which had submicromolar K_d_ values for all JNK isoforms. Introduction of a morpholinomethyl substituent at R^1^ led to compound **4m**, which had high water solubility and relatively high binding affinity for all JNKs. The enhanced solubility of **4m** is clearly due to the presence of the morpholine moiety, which can easily be protonated in aqueous solution. The other synthesized oximes had low water solubility. To increase their bioavailability for potential pharmaceutical applications, it will be necessary to apply special techniques (e.g., see [50]). Note that **4m** has been reported previously to be a potential antiviral agent and DNA intercalator **[51]**. Simultaneous substitution at R^3^ (Br or CF_3_) and R^1^ (CH_3_, CF_3_, Cl, or Br) led to inactive compounds **4c, 4h, 4i,** and **4j** for all JNK. Simultaneous substitution with two F atoms at R^3^ and R^2^ led to compound **4f** with high selectivity for JNK1/3 in comparison with JNK2. Simultaneous substitution at R^1^ and R^3^ with two F atoms led to compound **4e**, which also had high binding affinity (submicromolar K_d_) for JNK1 and JNK3. However, according to the ^1^H NMR data, the intermediate ketone for this compound is a mixture of two regioisomers of **3e** in a ratio of 63:37. There was no reason to assume that the corresponding oxime differed in isomeric composition, so the real affinity of the active isomer may be 1.5–3 times higher than that which we found.

Note that *Z*,*E*-isomerization of oximes can occur readily [52], hence isomerization is possible on interaction with a kinase binding site, as discussed previously [36]. Our calculation by the DFT method gives an estimated ΔG°_298_ value of 2.26 kJ/mol for *Z* ⇄ *E* isomerization of oxime **4f** (i.e., the *E*-isomer is just slightly less thermodynamically stable than the *Z*-isomer), and the *E*-configuration can be adopted by the molecule upon interaction with the JNK1-3 binding sites.

### 2.3. Evaluation of Compound Biological Activity

Prior to biological evaluation, we analyzed cytotoxicity of the compounds at various concentrations up to 50 μM in human monocytic THP-1Blue and MonoMac-6 cells during a 24-h incubation with the compounds. Compounds **4c**, **4j**, and **4l** exhibited cytotoxicity in THP1-Blue cells, and compounds **4c**, **4g**, and **4h** were cytotoxic in MonoMac-6 cells (Table 4). Thus, all six of these compounds were excluded from further biological evaluation.

**Figure 3:**
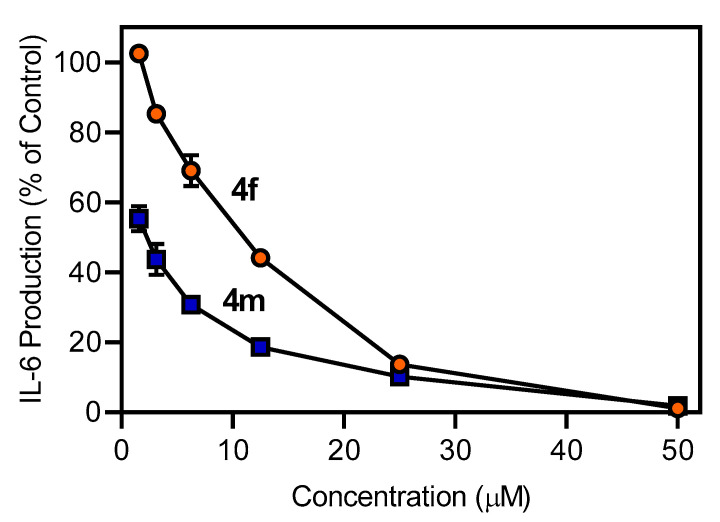
Effect of the compounds **4f** and **4m** on IL-6 production by human MonoMac-6 cells. MonoMac-6 cells were pretreated with the indicated compounds or DMSO (negative control) for 30 min followed by addition of 250 ng/mL LPS or buffer for 24 h. Production of IL-6 in the supernatants was evaluated by ELISA. The data are presented as the mean ± S.D. of triplicate samples from one experiment that is representative of three independent experiments.

The remaining non-cytotoxic oxime derivatives were evaluated for their ability to inhibit LPS-induced NF-κB/AP-1 reporter activity and interleukin (IL)-6 production in THP-1Blue and MonoMac-6 cells, respectively. As shown in Table 4, most of these compounds inhibited NF-κB/AP-1 reporter expression with activity that exceeded that of **IQ-1**, including a few compounds that did not have affinity (**4h** and **4i**) or only had very low affinity (**4k**) for JNK (see Table 3). Similarly, all compounds inhibited IL-6 production in MonoMac-6 cells (Table 4), including a few compounds that did not have affinity (**4i** and **4j**) or only had very low affinity (**4k**) for JNK (see Table 3). As an example, the dose-dependent inhibition of LPS-induced IL-6 production by compounds **4f** and **4m** is shown in Figure 3. The inhibitory activity of **4h**–**4k** may be due to interaction with non-JNK targets in these cells.

**Figure 4:**
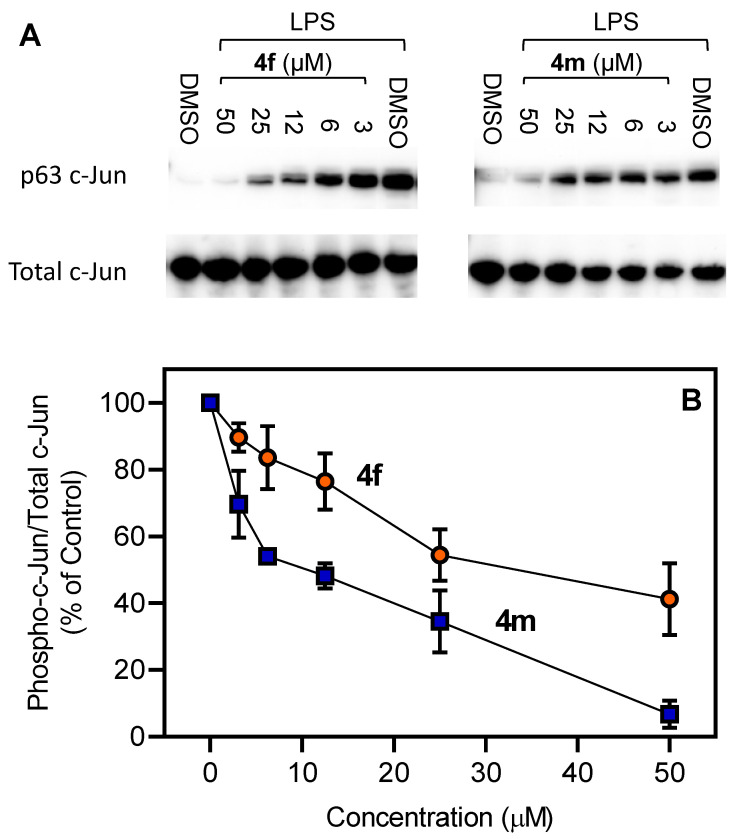
Effect of **4f** and **4m** on LPS-induced c-Jun (Ser63) phosphorylation. Human MonoMac-6 monocytic cells were pretreated with indicated concentrations of **4f** or **4m** for 30 min, followed by treatment with LPS (250 ng/mL) or control vehicle (1% DMSO) for another 30 min. The cells were lysed, and the lysates were analyzed by Western blotting. Total JNK (non-phosphorylated) was used as loading control for the lysates. A representative blot from two independent experiments is shown (**Panel A**). The blots were analyzed by densitometry, and the ratio of phospho-c-Jun/total c-Jun is shown in **Panel B**.

To confirm that the active compounds were inhibiting JNK activity, we selected two compounds (**4f** and **4m**) and evaluated their effects on c-Jun phosphorylation. MonoMac-6 monocytic cells were pretreated with the compounds, stimulated with LPS, and the level of phospho-c-Jun (Ser63) was determined. As expected, both compounds inhibited c-Jun phosphorylation in LPS-treated cells (Figure 4), verifying that the active compounds did indeed inhibit c-Jun phosphorylation at a similar concentration range as their inhibitory effects on IL-6 production in MonoMac-6 cells.

### 2.4. ADME Predictions

The ADME properties determining either the access of a potential drug to the target or its elimination by the organism are necessary during initial stages of drug discovery [53]. We evaluated ADME characteristics of the most potent compounds (**4b**, **4f**, and **4m**) using the SwissADME online tool [54]. As shown in Table 5, ADME assessment predicts that all three of these compounds would permeate through the blood–brain barrier, which is important since JNK3 is primarily located in the central nervous system.

**Figure 5:**
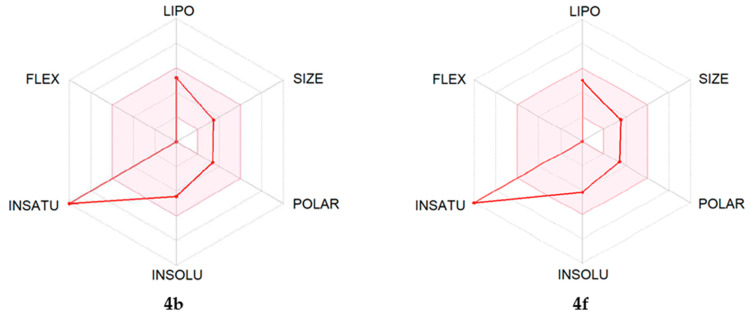

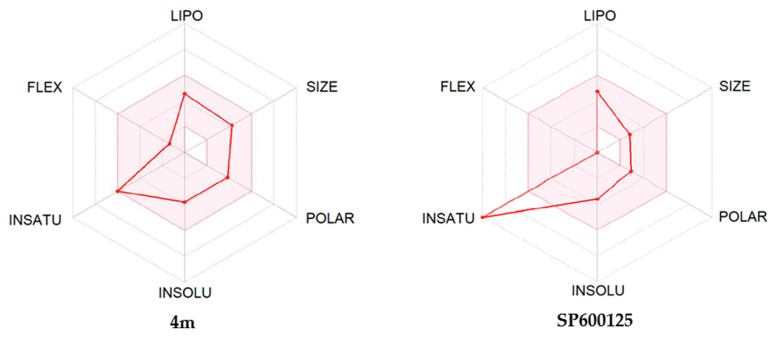
Bioavailability radar plots for compounds **4b**, **4f**, **4m**, and **SP600125**. The plots depict LIPO (lipophilicity), SIZE (molecular weight), POLAR (polarity), INSOLU (insolubility), INSATU (unsaturation), and FLEX (rotatable bond flexibility) parameters.

We also created bioavailability radar plots that display assessment of the drug-likeness of compounds **4b**, **4f**, and **4m**. Six physicochemical properties were considered: lipophilicity (LIPO), size, polarity (POLAR), insolubility (INSOLU), flexibility (FLEX), and unsaturation (INSATU). The physicochemical range on each axis is depicted as a pink area in which the radar plot of the molecule must fall entirely to have high predicted bioavailability [54]. We found that these heterocyclic oximes in general had satisfactory ADME properties, and the plots suggest high bioavailability (Figure 5). The only unfavorable property was a high unsaturation score in compounds **4b** and **4f**, which is common for most 11*H*-indeno[1,2-*b*]quinoxalin-11-one derivatives. Notably, the anthrapyrazolone JNK inhibitor **SP600125** [32] also has high unsaturation and a bioavailability radar very similar to compounds **4b** and **4f**. The morpholine derivative **4m** contains more aliphatic CH_2_ groups and thus has a ‘better’ bioavailability radar (Figure 5) and lower lipophilicity (Table 5). Additionally, the morpholine moiety can easily be N-protonated with salt formation, which usually improves drug delivery properties.

## 3. Conclusions

Synthesis and analysis of novel 11*H*-indeno[1,2-*b*]quinoxalin-11-one oxime analogs demonstrated that several of these compounds had high affinity for JNK1-3. These analogs also inhibited LPS-induced nuclear NF-κB/AP-1 activation and IL-6 production in human monocytic cells. In silico evaluation of ADME characteristics indicated that **4f** can cross the blood–brain barrier and thus has potential for use in the treatment of neuroinflammation and ischemia-reperfusion injury. Finally, the identified oximes represent new chemical tools that may be useful in further development of JNK inhibitors and could find application in the treatment of inflammatory diseases and neurodegenerative pathologies.

## 4. Experimental Section

### 4.1. Chemistry

Reaction progress was monitored by thin-layer chromatography (TLC) with UV detection using pre-coated silica gel F254 plates (Merck) or a Silufol UV-254. The synthesized structures were confirmed on the basis of analytical and spectral data. The melting points (m.p.) were determined using an electrothermal Mel-Temp capillary melting point apparatus. LC-HRMS analysis was performed on an Agilent G6538A 6538 UHD Accurate-Mass Q-TOF LC/MS (800-3728) instrument. NMR spectra were recorded on Bruker spectrometers (600 MHz for ^1^H, 150 MHz for ^13^C, and 280 MHz for ^19^F NMR). ^1^H, ^13^C, and ^19^F NMR spectra for the most potent compounds **4a**, **4b**, **4e**, **4f**, and **4m** are shown in Supplementary Materials.

#### 4.1.1. General Procedures for Indenoquinoxalin-11-one (**3a**–**3o**) Synthesis

A hot solution of 1 mmol of ninhydrin in 0.5 mL of acetic acid was added to a hot solution of 1 mmol of the appropriate substituted 1,2-diaminobenzene in 0.2 mL of acetic acid, stirred, and left for 1 h at 100 °C. Hot H_2_O (0.7 mL) was added to the reaction mixture, stirred, and left overnight. The precipitate was separated by centrifugation, washed three times with 10% acetic acid until neutral and dried under vacuum over NaOH. The dry crude product was dissolved in CHCl_3_, the solution was filtered through a layer of silica gel G (2 × 4 cm), the sorbent was washed with CHCl_3_ while controlling the composition of the eluate by TLC, and the filtrate was evaporated under vacuum to dryness to obtain the corresponding substituted indenoquinoxalin-11-ones.

*7-Fluoro-11H-indeno[1,2-b]quinoxalin-11-one* (**3a**). Yield 16%; M.p. 253–254 °C. ^1^H NMR (600 MHz, CDCl_3_) δ ppm: 7.5 (t, *J* = 8.4 Hz, 1 H), 7.6 (t, *J* = 7.5 Hz, 1 H), 7.7 (m, 2 H) 7.9 (d, *J* = 7.3 Hz, 1 H), 8.0 (d, *J* = 7.5 Hz, 1 H), 8.2 (m, 1 H); C_15_H_7_FN_2_O. M.W. 250.23. ESI-MS *m*/*z* (I%): 251(100%).

*6-Chloro-11H-indeno[1,2-b]quinoxalin-11-one* (**3b**). Yield 41%; M.p. 262–263 °C. ^1^H NMR (CDCl_3_) δ ppm: 7.6 (t, *J* = 7.5 Hz, 1 H), 7.6 (t, *J* = 8.0 Hz, 1 H), 7.7 (t, *J* = 7.5 Hz, 1 H), 7.8 (dd, *J* = 7.7, 1.1 Hz, 1 H), 7.9 (d, *J* = 7.5 Hz, 1 H), 8.1 (dd, *J* = 8.3, 1.1 Hz, 1 H), 8.2 (d, *J* = 7.5 Hz, 1 H). C_15_H_7_ClN_2_O. M.W. 266.69. ESI-MS: 268(40%), 266(100%), 238(30%).

*6-Methyl-8-bromo-11H-indeno[1,2-b]quinoxalin-11-one* (**3c**). Yield 18%; M.p. 200–201 °C. ^1^H NMR (CDCl_3_) δ ppm: 2.747 (s, 3 H), 7.530 (t, *J* = 7.519 Hz, 1 H), 7.679 (m, 1 H), 7.698 (m, 1 H), 7.842 (d, *J* = 7.520 Hz, 1 H), 8.028 (d, *J* = 7.520 Hz, 1 H), 8.142 (d, *J* = 1.651 Hz, 1 H). C_16_H_9_BrN_2_O. M.W. 325.17. ESI-MS: 325(15%), 327(15%).

*7-tert-Butyl-11H-indeno[1,2-b]quinoxalin-11-one* (**3d**). Yield 45%; M.p. 130–131 °C. ^1^H NMR (CDCl_3_) δ ppm: 1.4 (d, *J* = 11.0 Hz, 18 H), 7.5 (q, *J* = 7.2 Hz, 2 H), 7.7 (td, *J* = 7.5, 3.9 Hz, 2 H), 7.8 (dd, *J* = 8.8, 2.0 Hz, 1 H), 7.8 (m, 3 H), 8.0 (d, *J* = 8.8 Hz, 1 H), 8.0 (m, 3 H), 8.1 (d, *J* = 8.8 Hz, 1 H), 8.1 (d, *J* = 2.0 Hz, 1 H). C_19_H_16_N_2_O. M.W. 288.35. ESI-MS: 289(100%).

*6,8-Difluoro-11H-indeno[1,2-b]quinoxalin-11-one* (**3e**). Yield 45%; M.p. 200–201 °C. 1H NMR (CDCl_3_) δ ppm: 7.2 (m, 1 H), 7.5 (m, 1 H), 7.6 (m, 1 H), 7.7 (t, *J* = 7.5 Hz, 1 H), 7.9 (t, *J* = 6.7 Hz, 1 H), 8.0 (d, *J* = 7.5 Hz, 1 H). C_15_H_6_F_2_N_2_O. M.W. 268.22. ESI-MS: 269(100%).

*7,8-Difluoro-11H-indeno[1,2-b]quinoxalin-11-one* (**3f**). Yield 66%; M.p. 249–250 °C. 1H NMR (CDCl_3_) δ ppm: 7.56 (t, *J* = 7.43 Hz, 1 H), 7.72 (t, *J* = 7.52 Hz, 1 H), 7.81 (dd, *J* = 10.36, 7.98 Hz, 1 H), 7.86 (d, *J* = 7.52 Hz, 1 H), 7.92 (dd, *J* = 9.90, 8.25 Hz, 1 H), 8.01 (d, *J* = 7.52 Hz, 1 H). C_15_H_6_F_2_N_2_O. M.W. 268.22. ESI-MS: 269(100%).

*6,7-Diluoro-11H-indeno[1,2-b]quinoxalin-11-one* (**3g**). Yield 41%; M.p. 285–286 °C. ^1^H NMR (CDCl_3_) δ ppm: 7.6 (m, 1 H), 7.6 (t, *J* = 7.5 Hz, 1 H), 7.7 (t, *J* = 7.6 Hz, 1 H), 7.9 (d, *J* = 7.5 Hz, 1 H), 8.0 (ddd, *J* = 9.2, 5.0, 1.8 Hz, 1 H), 8.2 (d, *J* = 7.7 Hz, 1 H). C_15_H_6_F_2_N_2_O. M.W. 268.22. ESI-MS: 269(100%).

*6-Bromo-8-trifluoro-11H-indeno[1,2-b]quinoxalin-11-one* (**3h**). Yield 40%; M.p. 175–176 °C. ^1^H NMR (CDCl_3_) δ ppm: 7.73 (t, *J* = 7.52 Hz, 1 H), 7.87 (t, *J* = 7.52 Hz, 1 H), 8.01 (d, *J* = 7.52 Hz, 1 H), 8.29 (d, *J* = 7.52 Hz, 1 H), 8.31 (d, *J* = 1.47 Hz, 1 H), 8.51 (s, 1 H). C_16_H_6_BrF_3_N_2_O. M.W. 379.14. ESI-MS: 380(100%), 378(90%).

*6-Chloro-8-trifluoro-11H-indeno[1,2-b]quinoxalin-11-one* (**3i**). Yield 42%; M.p. 157–158 °C. ^1^H NMR (CDCl_3_) δ ppm: 7.7 (t, *J* = 7.4 Hz, 1 H), 7.9 (t, *J* = 7.5 Hz, 1 H), 8.0 (d, *J* = 7.5 Hz, 1 H), 8.1 (d, *J* = 1.3 Hz, 1 H), 8.2 (d, *J* = 7.5 Hz, 1 H), 8.4 (s, 1 H). C_16_H_6_ClF_3_N_2_O. M.W. 334.69. ESI-MS: 336(40%), 334(100%), 306(40%).

*6,8-bis(Trifluoromethyl)-11H-indeno[1,2-b]quinoxalin-11-one* (**3j**). Yield 81%; M.p. 171–172 °C. ^1^H NMR (CDCl_3_) δ ppm: 7.64 (ddd, *J* = 7.52, 0.92 Hz, 1 H), 7.78 (ddd, *J* = 7.52, 0.73 Hz, 1 H), 7.90 (d, *J* = 7.52 Hz, 1 H), 8.15 (d, *J* = 7.52 Hz, 1 H), 8.23 (s, 1 H), 8.60 (s, 1 H). C_17_H_6_F_6_N_2_O. M.W. 368.24. ESI-MS: 368(100%), 340(60%).

*8-Chloro-7-fluoro-11H-indeno[1,2-b]quinoxalin-11-one* (**3k**). Yield 42%; M.p. 136–137 °C. ^1^H NMR (CDCl_3_) δ ppm: 7.65 (m, 2 H), 7.86 (m, 2 H), 8.19 (s, 1 H), 8.30 (s, 1 H), C_15_H_6_F_Cl_N_2_O. M.W. 284.68. ESI-MS: 286(30%), 284(100%), 256(20%).

For methyl 11-oxo-11*H*-indeno[1,2-*b*]quinoxaline-8-carboxylate (**3l**) [39], 1 g (3.6 mmol) of **3n** was suspended in 5 mL of dry dimethylformamide, and 0.88 g (5.4 mmol) 1,1′- carbonyldiimidazole was added at 40 °C. The mixture was stirred at this temperature for 30 min and cooled to 20 °C. A solution of 0.390 g (7.2 mmol) of sodium methylate in 1 mL of methanol was added, and the mixture was stirred for 15 min. Most of the solvent was removed under reduced pressure, 50 mL of a 5% solution of Na_2_CO_3_ was added, and the mixture was extracted with CHCl_3_. The extract was washed (3 × 10 mL) with a 5% solution of Na_2_CO_3_, three times with H_2_O, dried over magnesium sulfate, and filtered through a column of silica gel (2 × 4 cm) while controlling the composition of the eluate by TLC. Yield 57%; M.p. 270–271 open (294–295 in cap.) °C. ^1^H NMR (CDCl_3_) δ ppm: 4.0 (s, 3 H), 7.6 (t, *J* = 7.3 Hz, 1 H), 7.7 (t, *J* = 7.5 Hz, 1 H), 7.9 (d, *J* = 7.5 Hz, 1 H), 8.1 (t, *J* = 8.6 Hz, 1 H), 8.3 (dd, *J* = 8.8, 1.8 Hz, 1 H), 8.8 (d, *J* = 1.7 Hz, 1 H). C_17_H_10_N_2_O_3_. M.W. 290.28. ESI-MS: 290(90%), 259(100%).

*6-(Morpholinomethyl)-11H-indeno[1,2-b]quinoxalin-11-one* (**3m**). Yield 46%; M.p. 236–237 °C. ^1^H NMR (CDCl_3_) δ ppm: 2.6 (s, 4 H), 3.7 (s, 4 H), 4.2 (s, 2 H), 7.5 (t, *J* = 7.4 Hz, 1 H), 7.7 (t, *J* = 7.6 Hz, 1 H), 7.7 (t, *J* = 7.4 Hz, 1 H), 7.9 (d, *J* = 7.5 Hz, 1 H), 7.9 (d, *J* = 5.3 Hz, 1 H), 8.1 (d, *J* = 7.3 Hz, 1 H), 8.1 (d, *J* = 8.3 Hz, 1 H). C_20_H_17_N_3_O_2_. M.W. 331.38. ESI-MS: 332(45%), 247(100%).

*11-Oxo-11H-indeno[1,2-b]quinoxaline-8-carboxylic Acid* (**3n**) [43]. Yield 68%; M.p. > 320 °C. C_16_H_8_F_6_N_2_O_3_. M.W. 276.25. ESI-MS: 276(100%), 248(40%), 231(15%).

*6-Methyl-11H-indeno[1,2-b]quinoxalin-11-one* (**3o**) [43]. Yield 83%; M.p. 226–227 °C. C_16_H_10_N_2_O. M.W. 246.27. ESI-MS: 246(80%), 232(30%), 218(20%), 43(100%).

*6-(Bromomethyl)-11H-indeno[1,2-b]quinoxalin-11-one* (**3p**) [44]. Yield 89%; M.p. 245–246 °C. C_16_H_9_BrN_2_O. M.W. 325.15. ESI-MS: 326(45%), 324(65%), 245(100%).

#### 4.1.2. General Procedure for Oximation of the Indenoquinoxaline Ketones

A solution of 1.5 mmol of hydroxylamine hydrochloride in a mixture of 0.3 mL of pyridine and 0.3 mL of ethanol was added to a solution of 0.3 mmol of the appropriate substituted indenoquinoxalin-11-one in 0.5 mL of pyridine and heated at 100 °C for 6 h. Next, 9 mL of 10% acetic acid solution was added, and the mixture was left overnight. The precipitate was centrifuged, triple washed with a 5% solution of acetic acid until neutral and dried under vacuum over NaOH. The crude products were recrystallized from dimethylacetamide, ethanol, or a mixture thereof.

*7-Fluoro-11H-indeno[1,2-b]quinoxalin-11-one Oxime* (**4a**). Yield 35%; M.p. 250–251 °C. ^1^H NMR (DMSO-d_6_) δ ppm: 7.7 (dd, 5.5 + 2.4 Hz 2 H), 7.8 (d, 8.8 Hz 2 H), 8.0 (dd, 7.5 + 1.1 Hz 1 H), 8.1 (dd, 8.2 + 1.1 Hz 1 H), 8.2 (m, 1 H), 8.6 (m, 1 H),13.4 (s, 1 H). ^13^C NMR (DMSO-d_6_) δ ppm: 122.8, 129.0, 129.5, 130.0, 130.6, 132.4, 132.6, 133.2, 133.7, 136.0, 138.7, 143.0, 147.2, 151.9, ESI-MS: [M − H]^−^ Calcd for C_15_H_8_FN_3_O 264.0573; Found 264.0565.

*6-Chloro-11H-indeno[1,2-b]quinoxalin-11-one Oxime* (**4b**). Yield 35%; M.p. 270–271 °C. ^1^H NMR (DMSO-d_6_) δ ppm: 7.7 (m, 3 H), 7.9 (m, 1 H), 8.2 (m, 2 H), 8.6 (m, 1 H), 13.4 (s, 1 H). ^13^C NMR (DMSO-d_6_) δ ppm: 113.6, 119.9, 122.7, 129.0, 132.3, 133.1, 133.6, 136.0, 139.1, 143.0, 147.3, 150.7, 153.9, 161.9, 163.5. ^19^F NMR (DMSO-d_6_) δ ppm: −108.7. ESI-MS: [M − H]^−^ Calcd for C_15_H_8_ClN_3_O 280.0278; Found 280.0271.

*6-Methyl-8-bromo-11H-indeno[1,2-b]quinoxalin-11-one Oxime* (**4c**). Yield 35%; M.p. 255–256 °C. ^1^H NMR (DMSO-d_6_) δ ppm: 2.8 (s, 3 H), 7.7 (m, 1 H), 7.8 (m, 1 H), 8.1 (m, 1 H), 8.5 (m, 1 H), 13.4 (s, 1 H). ^13^C NMR (DMSO-d_6_) δ ppm: 17.1, 122.3, 122.5, 128.9, 129.7, 132.2, 132.7, 133.3, 133.3, 136.1, 139.9, 140.0, 142.6, 147.25, 151.6, 152.3. ESI-MS: [M − H]^−^ Calcd for C_16_H_10_BrN_3_O 337.9929; Found 337.9914.

(*11Z/E)-7-tert-Butyl-11H-indeno[1,2-b]quinoxalin-11-one Oxime* (**4d**). Yield 35%; M.p. 197–198 °C. ^1^H NMR (DMSO-d_6_) δ ppm: 1.4 (s, 9 H), 7.7 (m, 2 H), 7.9 (m, 2 H), 8.0 (m, 1 H), 8.1 (m, 1 H), 8.2 (m, *J* = 7.0 Hz, 1 H), 8.5 (m, *J* = 6.8 Hz, 1 H), 13.32 (s, 0.45 H), 13.34 (s, 0.40 H). ^13^C NMR (DMSO-d_6_) δ ppm: 31.3, 35.4, 35.4, 122.3, 124.9, 125.4, 128.8, 129.1, 129.4, 129.6, 132.3, 132.5, 132.6, 133.2, 133.3, 136.5, 140.2, 140.5, 141.7, 142.1, 147.5, 147.6, 150.6, 151.0, 152.8, 153.0, 153.2, 153.7. ESI-MS: [M − H]^−^ Calcd for C_19_H_17_N_3_O 302.1293; Found 302.1282.

*6,8-Difluoro-11H-indeno[1,2-b]quinoxalin-11-one Oxime* (**4e**). Yield 35%; M.p. 255–256 °C. ^1^H NMR (DMSO-d_6_) δ ppm: 7.7 (m, 4 H), 8.1 (m, 1 H), 8.5 (m, 1 H), 13.4 (s, 0.5 H), 13.5 (s, 0.3 H). ^13^C NMR (DMSO-d_6_) δ ppm: 105.9, 106.1, 106.4, 106.6, 109.9, 110.4, 122.6, 122.8, 128.9, 129.8, 132.3, 133.0, 133.4, 133.7, 135.5, 135.6, 143.0, 143.4, 147.0, 150.6, 152.7, 152.9, 154.7, 157.1, 157.5, 159.2, 160.5, 161.0, 162.2, 162.7. ^19^F NMR (DMSO-Dd_6_) δ ppm: −120.8, −120.4, −107.5, −106.5. ESI-MS: [M − H]^−^ Calcd for C_15_H_7_F_2_N_3_O 282.0479; Found 282.0472.

*7,8-Difluoro-11H-indeno[1,2-b]quinoxalin-11-one Oxime* (**4f**). Yield 35%; M.p. 250–251 °C. ^1^H NMR (DMSO-d_6_) δ ppm: 7.7 (m, 2 H), 8.1 (dd, *J* = 5.6, 3.0 Hz, 1 H), 8.2 (m, 2 H), 8.5 (dd, *J* = 5.4, 3.2 Hz, 1 H), 13.4 (m, 1 H). ^13^C NMR (DMSO-d_6_) δ ppm: 115.7, 116.3, 122.5, 129.0, 132.3, 133.0, 133.2, 135.8, 139.2, 139.6, 147.2, 150.4, 150.8, 151.4, 152.1, 152.5, 153.5. ^19^F NMR (DMSO-d_6_) δ ppm: −132.4, −131.5. ESI-MS: [M − H]^−^ Calcd for C_15_H_7_F_2_N_3_O 282.0479; Found 282.0472.

*6,7-Difluoro-11H-indeno[1,2-b]quinoxalin-11-one Oxime* (**4g**). Yield 35%; M.p. 257–258 °C. ^1^H NMR (DMSO-d_6_) δ ppm: 7.7 (m, 2 H), 7.9 (m, 1 H), 8.0 (m, 1 H), 8.2 (m, *J* = 6.2 Hz, 1 H), 8.5 (m, *J* = 5.7, 2.0 Hz, 1 H), 13.5 (m, 1 H). ^13^C NMR (DMSO-d_6_) δ ppm: 119.6, 119.7, 119.7, 121.5, 123.0, 126.4, 129.0, 132.4, 133.2, 133.5, 133.8, 135.6, 139.1, 143.4, 143.5, 145.1, 145.2, 147.0, 147.0, 148.7, 148.8, 150.3, 150.4, 151.5, 153.7. ESI-MS: [M − H]^−^ Calcd for C_15_H_7_F_2_N_3_O 282.0479; Found 282.0465.

*6-Bromo-8-(trifluoromethyl)-11H-indeno[1,2-b]quinoxalin-11-one Oxime* (**4h**). Yield 78%; M.p. 236–237 °C. ^1^H NMR (DMSO-d_6_) δ ppm: 7.8 (m, 2 H), 8.2 (d, *J* = 6.4 Hz, 1 H), 8.5 (s, 1 H), 8.5 (s, 1 H), 8.6 (d, *J* = 6.1 Hz, 1 H), 13.7 (s, 1 H). ^13^C NMR (DMSO-d_6_) δ ppm: 122.6, 123.3, 124.4, 125.6, 127.8, 129.0, 129.1, 129.9, 130.1, 132.5, 134.0, 134.3, 135.4, 141.5, 142.0, 146.9, 153.3, 155.8. ESI-MS: [M − H]^−^ Calcd for C_16_H_7_BrF_3_N_3_O 391.9646; Found 391.9633.

*(11Z/E)-6-Chloro-8-(trifluoromethyl)-11H-indeno[1,2-b]quinoxalin-11-one Oxime(***4i***).* Yield 42%; M.p. 324–325 °C. ^1^H NMR (DMSO-d_6_) δ ppm: 7.75–7.80 (m, 2H), 8.23 (d, *J* = 6.6 Hz, 1H), 8.3 (d, 1 H), 8.5 (d, 1 H), 8.6 (dd, 1 H), 13.54 (s, 0.1 H), 13.65 (s, 1.1 H). ESI-MS: [M − H]^−^ Calcd for C_16_H_7_ClF_3_N_3_O 348.0151; Found 348.0139.

*6,8-bis(Trifluoromethyl)-11H-indeno[1,2-b]quinoxalin-11-one oxime* (**4j**). Yield 35%; M.p. 253–254 °C. ^1^H NMR (DMSO-d_6_) δ ppm: 7.8 (m, 1 H), 7.8 (td, *J* = 7.4, 1.3 Hz, 1 H), 8.2 (d, *J* = 7.2 Hz, 1 H), 8.4 (s, 1 H), 8.6 (s, 1 H), 8.8 (s, 1 H), 13.7 (s, 1 H). ^13^C NMR (DMSO-d_6_) δ ppm: 122.6, 122.7, 123.3, 124.1, 124.4, 124.5, 128.3, 128.5, 128.7, 129.0, 132.6, 132.7, 134.2, 134.4, 135.2, 140.8, 141.5, 146.8, 153.5, 155.4. ESI-MS: [M − H]^−^ Calcd for C_17_H_7_F_6_N_3_O 382.0415; Found 382.0407.

*8-Chloro-7-fluoro-11H-indeno[1,2-b]quinoxalin-11-one Oxime* (**4k**). Yield 35%; M.p. 276–277 °C. ^1^H NMR (DMSO-d_6_) δ ppm: 7.7 (m, 2 H), 8.1 (m, 2 H), 8.3 (dd, *J* = 7.6, 2.8 Hz, 1 H), 8.5 (m, 1 H), 13.4 (s, 1 H). ^13^C NMR (DMSO-d_6_) δ ppm: 114.9, 115.0, 115.4, 115.5, 122.6, 122.8, 123.2, 123.3, 129.0, 130.7, 131.3, 132.4, 133.2, 133.3, 133.5, 133.6, 135.7, 139.1, 139.5, 141.4, 141.8, 147.1, 151.6, 152.2, 153.7, 154.2, 156.4, 156.8, 158.0, 158.4. ESI-MS: [M − H]^−^ Calcd for C_15_H_7_ClFN_3_O 298.0183; Found 298.0172.

*Methyl-11-(hydroxyimino)-11H-indeno[1,2-b]quinoxaline-8-carboxylate* (**4l**). Yield 35%; M.p. 300–301 °C. 1H NMR (DMSO-d_6_) δ ppm: 4.01 (s, 3 H), 7.80 (m, 2 H), 8.25 (m, 2 H), 8.29 (m, 1 H), 8.60 (m, *J* = 5.32, 3.48 Hz, 1 H), 8.64 (d, *J* = 1.83 Hz, 1 H), 13.55 (s, 1 H). ESI-MS: [M − H]^−^ Calcd for C_17_H_11_N_3_O_3_ 304.0722; Found 304.0715.

*6-(Morpholinomethyl)-11H-indeno[1,2-b]quinoxalin-11-one Oxime* (**4m**). A solution of 104 mg (1.5 mmol) of hydroxylamine hydrochloride in a mixture of 0.5 mL of H_2_O, 0.5 mL of ethanol, and 0.5 mL of a 3M NaOH solution was added sequentially to a solution of 100 mg (0.3 mmol) **3m** in 3 mL of ethanol. The mixture was heated in a boiling water bath, adding ethanol as necessary to maintain homogeneity of the reaction mixture until the **3m** spot on the sample chromatogram (silica gel plate, benzene: triethylamine 10:1) was absent. The reaction mixture was diluted with 10 mL of 0.2 M (NH_4_)_2_CO_3_ buffer solution (pH = 9). After cooling, the precipitate was centrifuged, washed with the same buffer solution (3 × 5 mL), distilled H_2_O (3 × 5 mL), dried under vacuum, and recrystallized from heptane to obtain 50 mg (48%) of **4m**. MP 210–211 °C. Yield 48%; M.p. 210–211 °C. ^1^H NMR (DMSO-d_6_) δ ppm: 2.6 (t, 4 H), 3.6 (t, 4 H), 4.2 (s, 2 H), 7.7 (m, 2 H), 7.8 (m, 1 H), 7.9 (d, *J* = 7.2 Hz, 1 H), 8.0 (d, *J* = 8.1 Hz, 1 H), 8.2 (d, 1 H), 8.5 (m, 1 H), 13.3 (s, 1 H). ^13^C NMR (DMSO-d_6_) δ ppm: 53.9, 56.6, 66.8, 122.4, 129.0, 129.0, 129.7, 130.4, 132.3, 132.7, 133.4, 136.5, 136.9, 140.9, 141.9, 147.5, 150.8, 152.1. ESI-MS: [M − H]^−^ Calcd for C_20_H_18_N_4_O_2_ 345.1351; Found 345.1346.

### 4.2. X-ray Diffraction Analysis

X-ray crystallography of **3b** crystals was performed on a Bruker Kappa Apex II CCD diffractometer using φ,ω-scans of narrow (0.5°) frames with Mo Kα radiation (λ = 0.71073 Å) and a graphite monochromator. The structures were solved by direct methods using the SHELX-97 program (University of Göttingen, Germany, 1997) and refined by full-matrix least-squares method against all F2 in anisotropic approximation using the SHELXL-2014/7 program [55]. Absorption corrections were applied using the empirical multiscan method with the SADABS program (Bruker AXS, Madison, WI, USA, 2008). The hydrogen atom positions were calculated with the riding model. Cambridge Crystallographic Data Centre 2080663 contains the supplementary crystallographic data for this paper, and these data can be obtained free of charge via http://www.ccdc.cam.ac.uk/cgi-bin/catreq.cgi (accessed on 1 August 2021) or from the Cambridge Crystallographic Data Centre, 12 Union Road, Cambridge CB2 1EZ, UK; deposit@ccdc.cam.ac.uk.

### 4.3. Methods for Biological Analysis

#### 4.3.1. Kinase *K*_d_ Determination

Selected compounds were submitted for dissociation constant (*K*_d_) determination using KINOMEscan (Eurofins Pharma Discovery, San Diego, CA, USA), as described previously [56]. In brief, kinases were produced and displayed on T7 phage or expressed in HEK-293 cells. Binding reactions were performed at room temperature for 1 h, and the fraction of kinase not bound to test compound was determined by capture with an immobilized affinity ligand and quantified by quantitative polymerase chain reaction. Primary screening at fixed concentrations of compounds was performed in duplicate. For dissociation constant *K*_d_ determination, a 12-point half-log dilution series (a maximum concentration of 33 μM) was used. Assays were performed in duplicate, and their mean value is displayed.

#### 4.3.2. Cell Culture

All cells were cultured at 37 °C in a humidified atmosphere containing 5% CO_2_. THP1-Blue cells obtained from InvivoGen (San Diego, CA, USA) were cultured in RPMI 1640 medium (Mediatech Inc., Herndon, VA, USA) supplemented with 10% (*v/v*) fetal bovine serum (FBS), 100 μg/mL streptomycin, 100 U/mL penicillin, 100 μg/mL phleomycin (Zeocin), and 10 μg/mL blasticidin S. Human monocyte-macrophage MonoMac-6 cells (Deutsche Sammlung von Mikroorganismen und Zellkulturen GmbH, Braunschweig, Germany) were grown in RPMI 1640 medium supplemented with 10% (*v/v*) FBS, 10 μg/mL bovine insulin, 100 μg/mL streptomycin, and 100 U/mL penicillin.

#### 4.3.3. Analysis of AP-1/NF-κB Activation

Activation of AP-1/NF-κB was measured using an alkaline phosphatase reporter gene assay in THP1-Blue cells. Human monocytic THP-1 Blue cells are stably transfected with a secreted embryonic alkaline phosphatase gene that is under the control of a promoter inducible by NF-κB/AP-1. THP1-Blue cells (2 × 10^5^ cells/well) were pretreated with test compound or DMSO for 30 min, followed by addition of 250 ng/mL lipopolysaccharide (LPS) for 24 h, and alkaline phosphatase activity was measured in cell supernatants using QUANTI-Blue mix (InvivoGen) as absorbance at 655 nm and compared with positive control samples (LPS). For selected compounds, the concentrations of inhibitor that caused 50% inhibition of the NF-κB reporter activity (IC_50_) were calculated.

#### 4.3.4. IL-6 Analysis

A human IL-6 ELISA kit (BD Biosciences, San Jose, CA, USA) was measure IL-6 production. MonoMac-6 cells were plated in 96-well plates at a density of 2 × 10^5^ cells/well in culture medium supplemented with 3% (*v*/*v*) endotoxin-free FBS. The cells were pretreated with test compound or DMSO for 30 min, followed by addition of 250 ng/mL LPS for 24 h. IC_50_ values for IL-6 production were calculated by plotting percentage inhibition against the logarithm of inhibitor concentration (at least five points).

#### 4.3.5. Cytotoxicity Assay

Cytotoxicity was analyzed with a CellTiter-Glo Luminescent Cell Viability Assay Kit from Promega (Madison, WI, USA), according to the manufacturer’s protocol. Cells were treated with compounds under investigation and incubated for 24 h. After treatment, the cells were equilibrated to room temperature for 30 min, substrate was added, and the samples were analyzed with a Fluoroscan Ascent FL (Thermo Fisher Scientific, Waltham, MA, USA). The IC_50_ values were calculated by plotting percentage inhibition against the logarithm of inhibitor concentration (at least five points).

#### 4.3.6. Western Blotting

MonoMac-6 monocytic cells were pretreated with different concentrations of the compounds under investigation for 30 min and treated with LPS (250 ng/mL) or vehicle for another 30 min. Cells were washed twice with Hanks’ balanced salt solution, and cell lysates were prepared using lysis buffer from the JNK kinase assay kit (Cell Signaling Technology, Danvers, MA). Cell lysates (from 5 × 10^6^ cells) were separated on ExpressPlus 4–20% PAGE Gels (GenScript, Piscataway, NJ, USA) using TRIS-MOPS running buffer (GenScript) and transferred to nitrocellulose membranes. The blots were probed with antibodies against c-Jun, phospho-c-Jun (Ser63), and total c-Jun (Cell Signaling Technology, Danvers, MA, USA), followed by horseradish peroxidase-conjugated secondary antibody (Cell Signaling Technology). The blots were developed using SuperSignal West Femto chemiluminescent substrate (Thermo Fisher Scientific) and visualized with a FluorChem FC2 imaging system (Alpha Innotech Corporation, San Leandro, CA, USA). Quantitation of the chemiluminescent signal was performed using AlphaView software (ver. 3.0; Alpha Innotech).

### 4.4. Molecular Modeling

DFT calculations were performed with the Gaussian 16 program (Gaussian, Inc., Wallingford CT) for compounds **2a**–**k**, **2n**, and *Z*, *E*-isomers of oxime **4f**. The hybrid B3LYP functional [57,58] and correlation consistent aug-cc-pVDZ basis set [59,60] were used. Vibrational frequency analysis was done for all the optimized geometries in order to ensure attaining energy minima for the molecules and to calculate thermodynamic properties of **4f** isomers.

The physicochemical properties of selected compounds were computed using SwissADME (http://www.swissadme.ch, accessed on 1 August 2021) [54].

## Figures and Tables

**Table 1:** Chemical structure and activity of previously identified JNK1-3 inhibitors with an 11*H*-indeno[1,2-*b*]quinoxalin-11-one scaffold containing different substituents in the tetracyclic moiety [35,36].

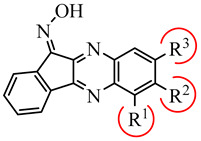				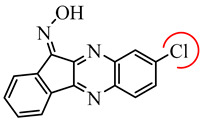			
Compound C							
	R^1^	R^2^	R^3^		K_d_ (µM)		
					JNK1	JNK2	JNK3
**IQ-1**	H	H	H		0.2	0.4	0.1
**A**	H	H	COOH		2.1	6.0	3.9
**B**	H	NO_2_	H		6.0	12.0	4.3
**C**	H	H	Cl		1.1	21.5	0.7
**D**	H	CH_3_	H		0.7	0.8	0.3
**E**	H	OC_2_H_5_	H		2.3	2.0	0.9
**F**	CH_3_	H	H		3.3	2.5	6.0
**G**	H	CH_3_	CH_3_		2.7	2.5	1.3

**Table 2:** Mulliken charges on nitrogen atoms of **2a–k**, **2n**.

Diamine	Mulliken Charges on Nitrogen Atoms ^a^	
	N1	N2
**2a**	−0.086	−0.072
**2b**	−0.117	−0.060
**2c**	−0.090	−0.097
**2d**	−0.033	−0.006
**2e**	−0.076	−0.118
**2f**	−0.077	−0.077
**2g**	−0.077	−0.092
**2h**	−0.107	0.015
**2i**	−0.100	−0.034
**2j**	−0.121	0.003
**2k**	−0.034	−0.084
**2n**	−0.101 ^b^	0.041 ^c^

^a^ Numbering of nitrogen atoms in the substituted benzenes corresponds to IUPAC rules; ^b^ charge on the nitrogen atom at position *3* of 3,4-diaminobenzoic acid; ^c^ charge on the nitrogen atom at position *4* of 3,4-diaminobenzoic acid.

**Table 3:**
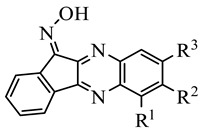
Chemical structures of synthesized oxime derivatives and JNK binding affinity.

Compound	R^1^	R^2^	R^3^	JNK1	JNK2	JNK3
				K_d_ (µM)		
IQ-1 *	H	H	H	0.24	0.36	0.10
**4a**	H	F	H	0.28 ± 0.07	0.62 ± 0.01	0.19 ± 0.01
**4b**	Cl	H	H	0.17 ± 0.04	0.22 ± 0.06	0.14 ± 0.03
**4c**	CH_3_	H	Br	N.B.	N.B.	N.B.
**4d**	H	t-Bu	H	3.1 ± 0.1	1.6 ± 0.6	3.1 ± 0.2
**4e**	F	H	F	0.92 ± 0.05	1.6 ± 0.01	0.9 ± 0.3
**4f**	H	F	F	0.17 ± 0.04	N.B.	0.52 ± 0.3
**4g**	F	F	H	0.52 ± 0.09	0.91 ± 0.06	1.1 ± 0.1
**4h**	Br	H	CF_3_	N.B.	N.B.	N.B.
**4i**	Cl	H	CF_3_	N.B.	N.B.	N.B.
**4j**	CF_3_	H	CF_3_	N.B.	N.B.	N.B.
**4k**	H	F	Cl	N.B.	N.B.	24.5 ± 2.1
**4l**	H	H	COOCH_3_	0.91 ± 0.16	2.4 ± 0.2	0.71 ± 0.05
**4m**	CH_2_MRF	H	H	1.1 ± 0.1	0.88 ± 0.10	0.91 ± 0.3

* Data for IQ-1 are from [35]. N.B., no binding affinity at concentrations < 30 μM. Abbreviations: MRF, morpholine.

**Table 4:** Effect of the compounds on LPS-induced NF-κB/AP-1 transcriptional activity in THP1-Blue cells, LPS-induced production of IL-6 in MonoMac-6 cells, and cytotoxicity.

Compound	THP-1Blue Cell Cytotoxicity	MonoMac-6 Cell Cytotoxicity	THP-1Blue Cell NF-κB/AP-1	MonoMac-6 Cell IL-6
			**IC_50_ (μM)**	
**IQ-1**	N.T	N.T	2.3 ± 0.8	3.4 ± 0.8
**4a**	N.T.	N.T.	0.3 ± 0.1	3.0 ± 0.6
**4b**	N.T.	N.T.	0.9 ± 0.3	1.2 ± 0.4
**4c**	37.5 ± 1.7	14.6 ± 1.2	n.d.	n.d.
**4d**	N.T.	N.T.	0.4 ± 0.1	1.0 ± 0.2
**4e**	N.T.	N.T.	0.4 ± 0.1	2.2 ± 0.2
**4f**	N.T.	N.T.	1.1 ± 0.4	14.0 ± 2.8
**4g**	N.T.	32.0 ± 2.2	2.1 ± 0.4	n.d.
**4h**	N.T.	29.0 ± 2.5	2.5 ± 0.6	n.d.
**4i**	N.T.	N.T.	2.9 ± 0.9	6.2 ± 0.2
**4j**	36.5 ± 1.9	N.T.	n.d.	4.8 ± 0.5
**4k**	N.T.	N.T.	0.6 ± 0.2	2.3 ± 0.4
**4l**	26.1 ± 5.3	N.T.	n.d.	N.A.
**4m**	N.T.	N.T.	0.1 ± 0.05	0.8 ± 0.1

N.A., no inhibition at concentrations < 50 μM; N.T., non-cytotoxic at concentrations < 50 μM; n.d., no data (activity was not evaluated because of compound cytotoxicity). Data for **IQ-1** are from [35].

**Table 5:** Predicted ADME properties of compounds **4b**, **4f**, and **4m**.

Property	4b	4f	4m
Formula	C_15_H_8_ClN_3_O	C_15_H_7_F_2_N_3_O	C_20_H_18_N_4_O_2_
Molecular Weight (g/mol)	281.70	283.23	346.38
Heavy Atoms	20	21	26
Fraction Csp3	0.00	0.00	0.25
Rotatable bonds	0	0	2
H-bond Acceptors	4	6	6
H-bond Donors	1	1	1
Molar Refractivity	77.41	72.32	102.38
Topological Polar Surface Area (tPSA, Å^2^)	58.37	58.37	70.84
Lipophilicity (Consensus Log P_o/w_)	3.08	3.16	2.39
BBB Permeation	Yes	Yes	Yes

## Data Availability

The data presented in this study are available on request from the corresponding author.

## Supplementary Materials

The following are available online, Figure S1: Crystal packing of compound **3b** along crystallographic axis *b*. Figures S2–S13: NMR spectral data for compounds **4a**, **4b**, **4e**, **4f**, and **4m**. Table S1: Bond lengths and angles of H-bonds for compound **3b** in the crystal.

## Sample Availability

Samples of the compounds are available from the authors.
