# Supplementary material for: Novel c-Jun N-Terminal Kinase (JNK) Inhibitors with an 11H-Indeno[1,2-b]quinoxalin-11-one Scaffold
Source: Molecules. 2021 Sep 20;26(18):5688. doi: 10.3390/molecules26185688 (PMC8464905; doi:10.3390/molecules26185688)
Supplement: Supplementary file 1 [file molecules-26-05688-s001.zip › molecules-1366857-supplementary.pdf]

Supplementary Material

For

Novel c-Jun N-terminal Kinase (JNK) Inhibitors with an 11*H*-Indeno[1,2-*b*]quinoxalin-11-one Scaffold

Serhii A. Liakhov[1], Igor A. Schepetkin[2], Olexander S. Karpenko[1], Hanna I. Duma[1], Nadiia M. Haidarzhy[3], Liliya N. Kirpotina[2], Anastasia R. Kovrizhina[4], Andrei I. Khlebnikov[4], Irina Yu. Bagryanskaya[5] and Mark T. Quinn[2]

[1]A.V. Bogatsky Physico-Chemical Institute, National Academy of Sciences of Ukraine, Odessa, Ukraine;
[2]Department of Microbiology and Cell Biology, Montana State University, Bozeman, MT 59717, USA;
[3] Odessa National Polytechnic University, Odessa, Ukraine;
[4]Kizhner Research Center, Tomsk Polytechnic University, Tomsk 634050, Russia;
[5]Vorozhtsov Institute of Organic Chemistry, Siberian Branch, Russian Academy of Sciences, Novosibirsk 630090, Russia

Supplementary Table S1. Bond lengths and angles of H-bonds for compound **3b** in the crystal[a].

		Distance (Å)		Angle[a]
**H-bond**	**D–H**	**H…A**	**D…A**	**D–H…A**
C9-H…N10	0.93	2.58	3.497(3)	168
C7′-H…O13	0.93	2.55	3.451(4)	163
C9′-H…N10′	0.93	2.58	3.493(4)	168

[a] Bond angles in degrees.

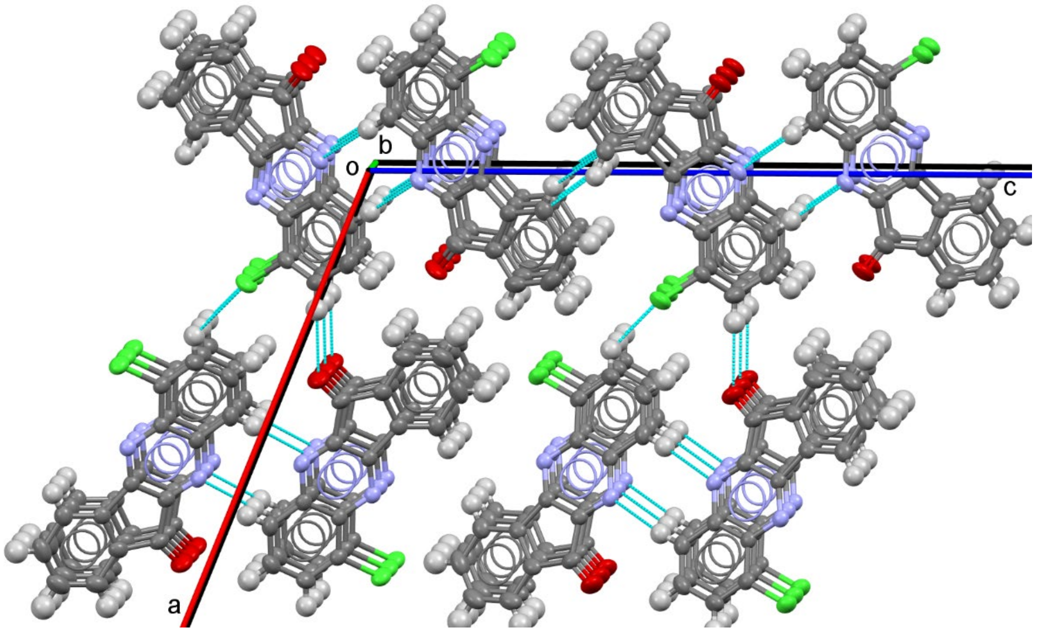

Supplementary Figure S1. Crystal packing of compound **3b** along crystallographic axis *b*. Oxygen and chlorine atoms are shown in red and green, respectively. Intermolecular H-bonds are shown as thin blue lines.

NMR Spectra Data

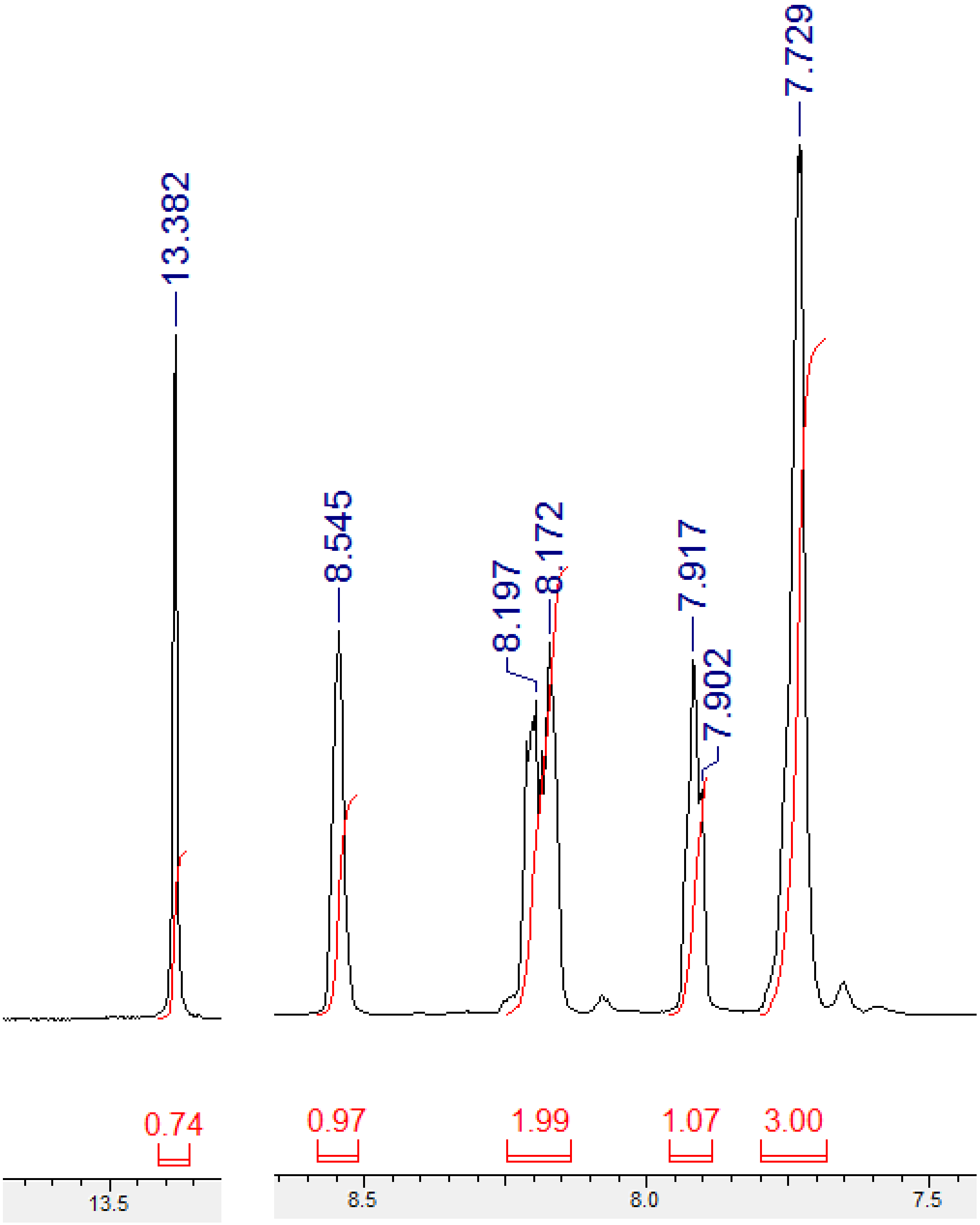

NMR ^{1}H spectrum (600 MHz, DMSO-D6) of compound **4a**.

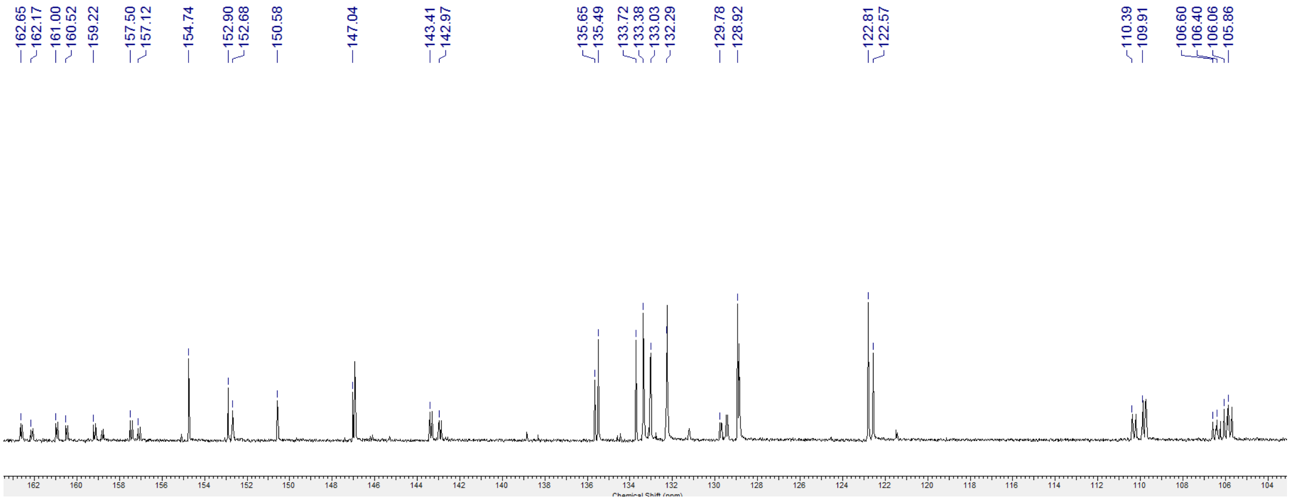

NMR ^{13}C spectrum (150 MHz, DMSO-D6) of compound **4a**.

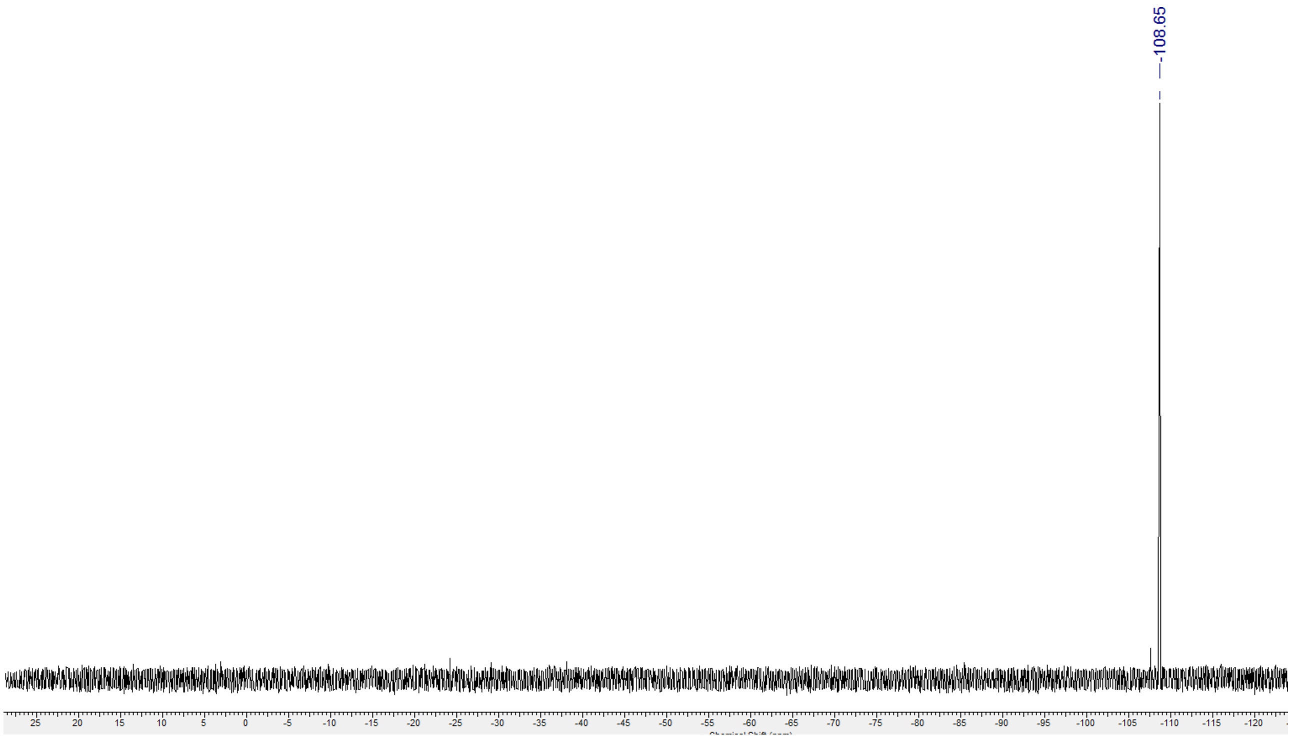

NMR ^{19}F spectrum (280 MHz, DMSO-D6) of compound **4a**.

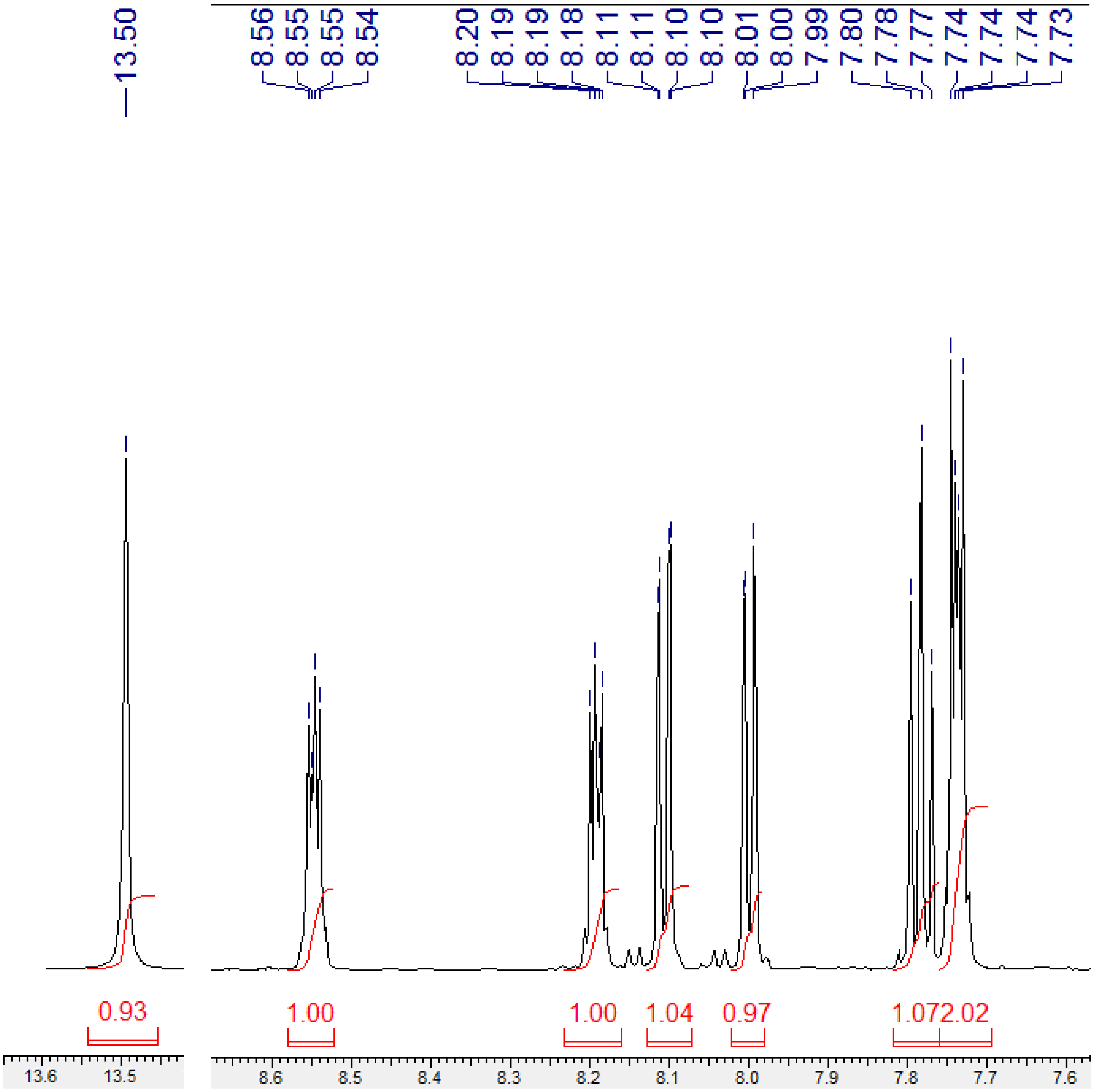

NMR ^{1}H spectrum (600 MHz, DMSO-D6) of compound **4b**.

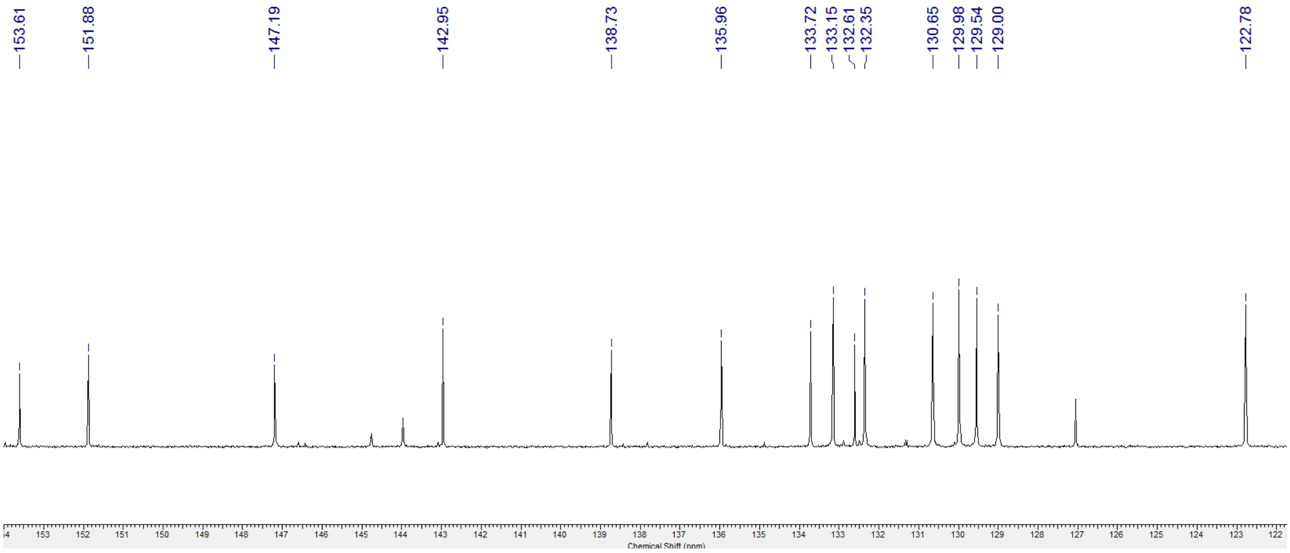

NMR ^{13}C spectrum (150 MHz, DMSO-D6) of compound **4b**.

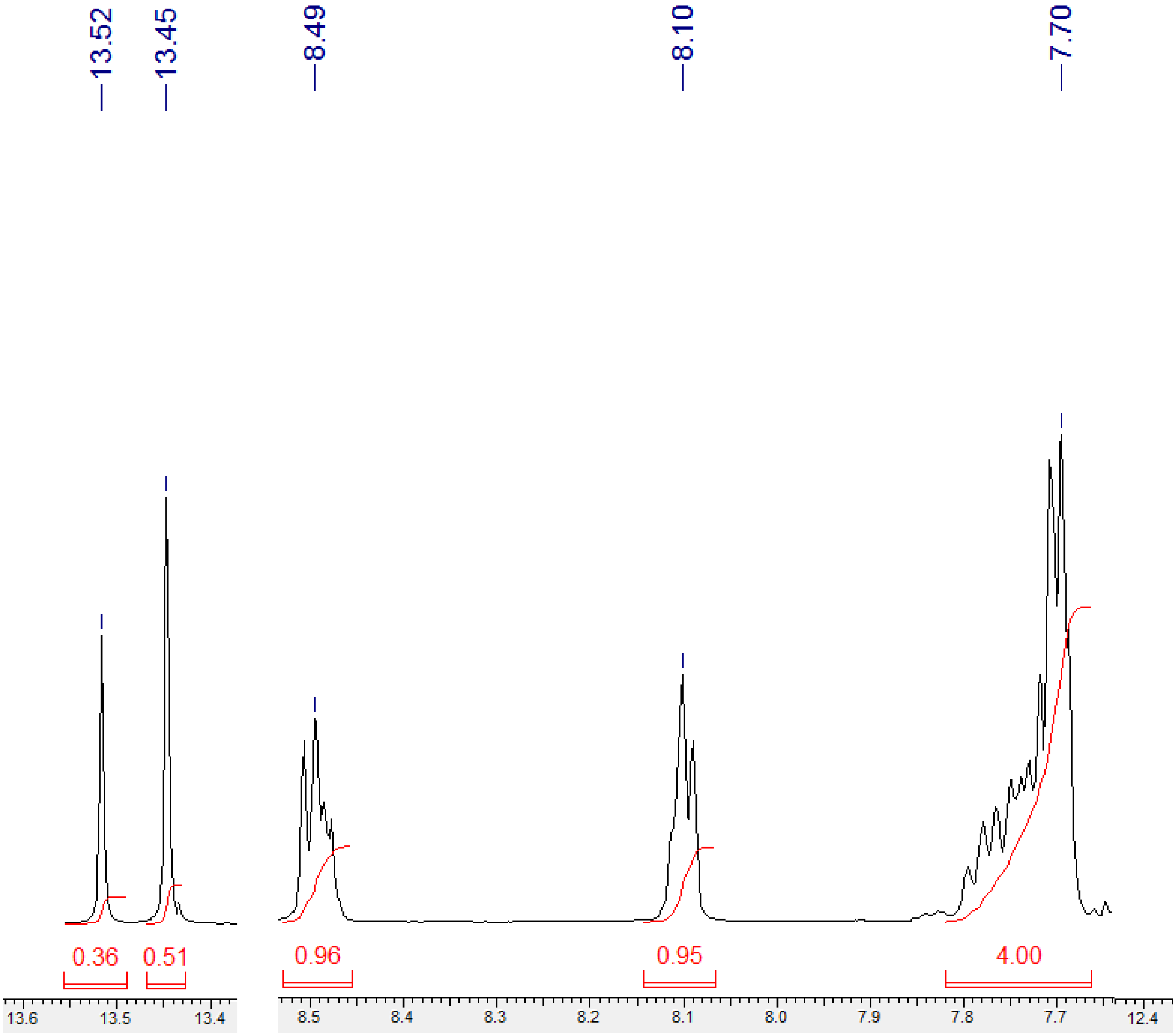

NMR ^{1}H spectrum (600 MHz, DMSO-D6) of compound **4e**.

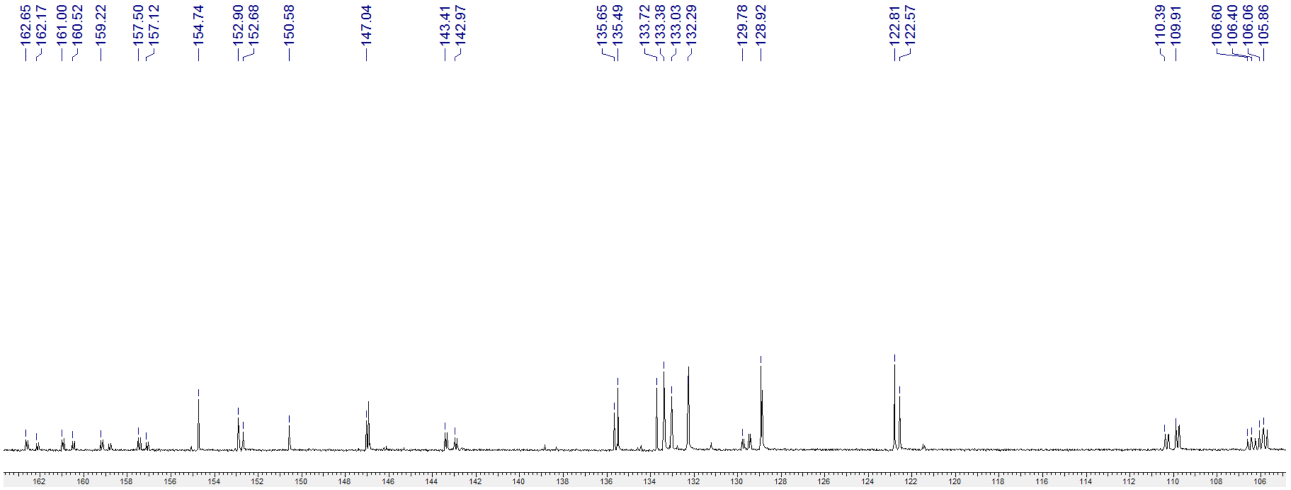

NMR ^{13}C spectrum (150 MHz, DMSO-D6) of compound **4e**.

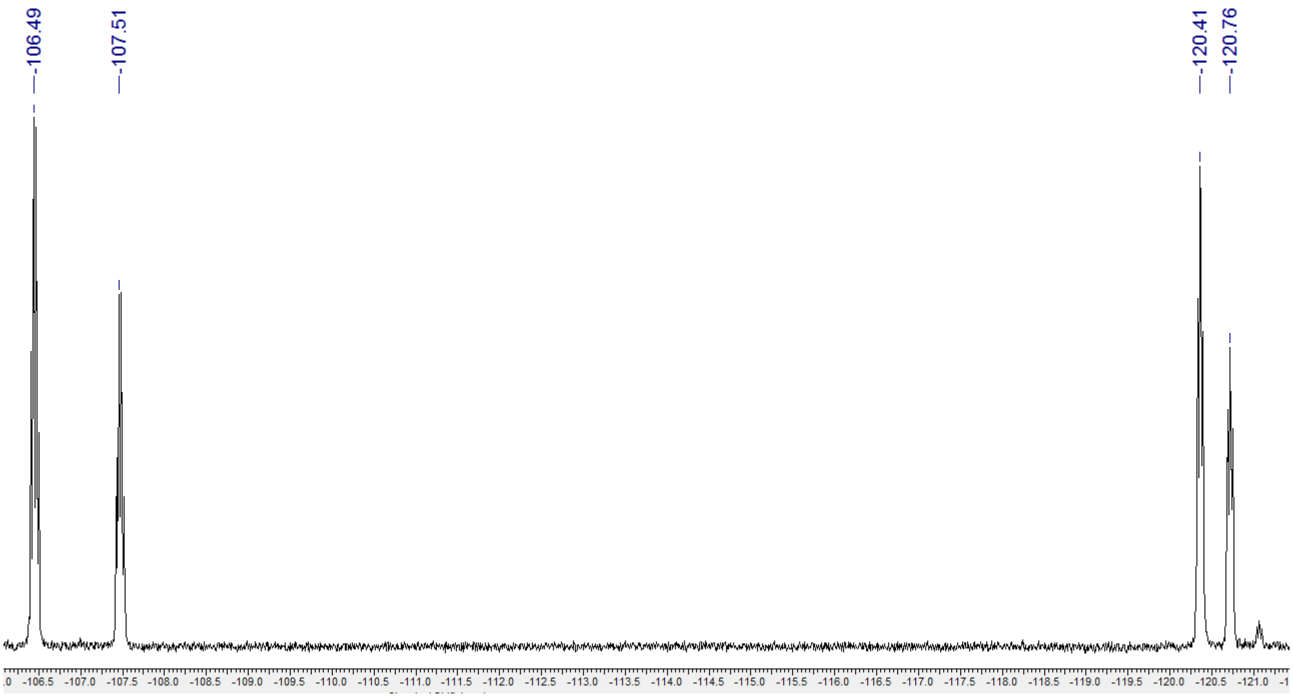

NMR ^{19}F spectrum (280 MHz, DMSO-D6) of compound **4e**.

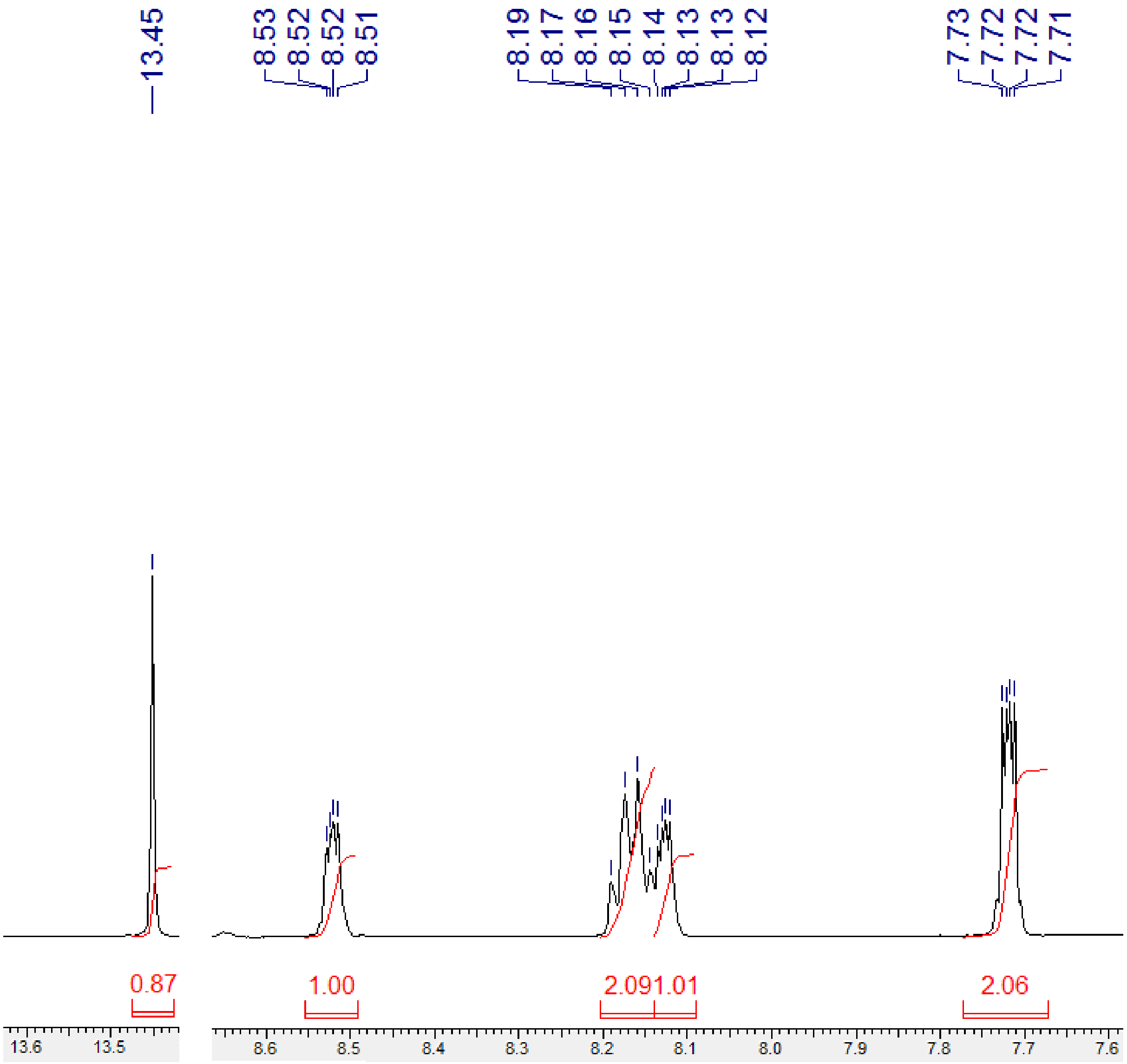

NMR ^{1}H spectrum (600 MHz, DMSO-D6) of compound **4f**.

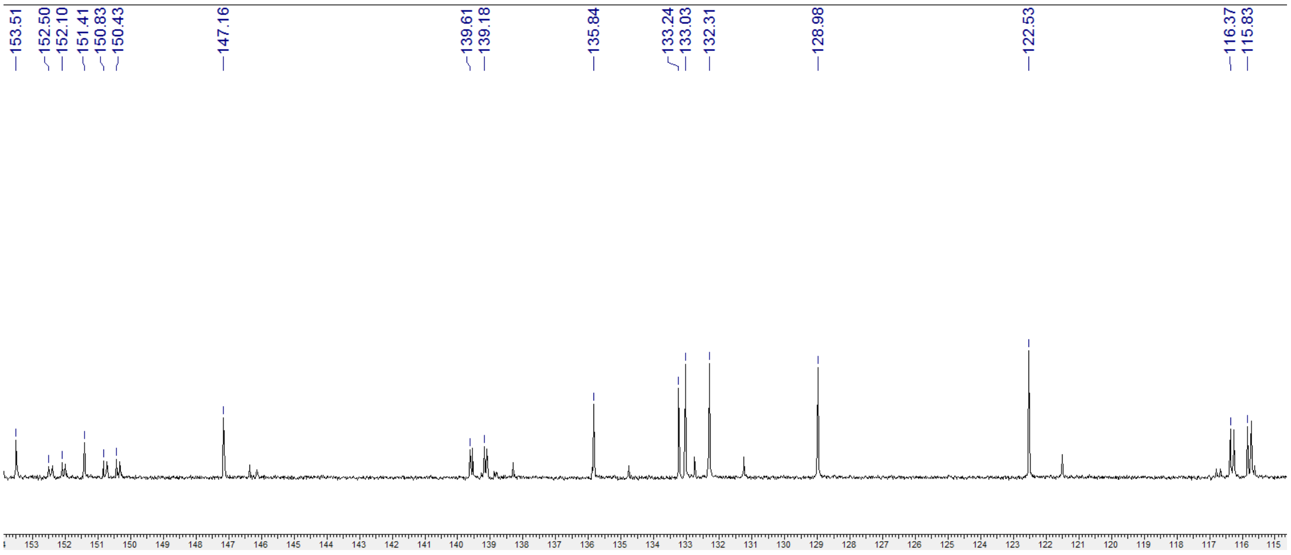

NMR ^{13}C spectrum (150 MHz, DMSO-D6) of compound **4f**.

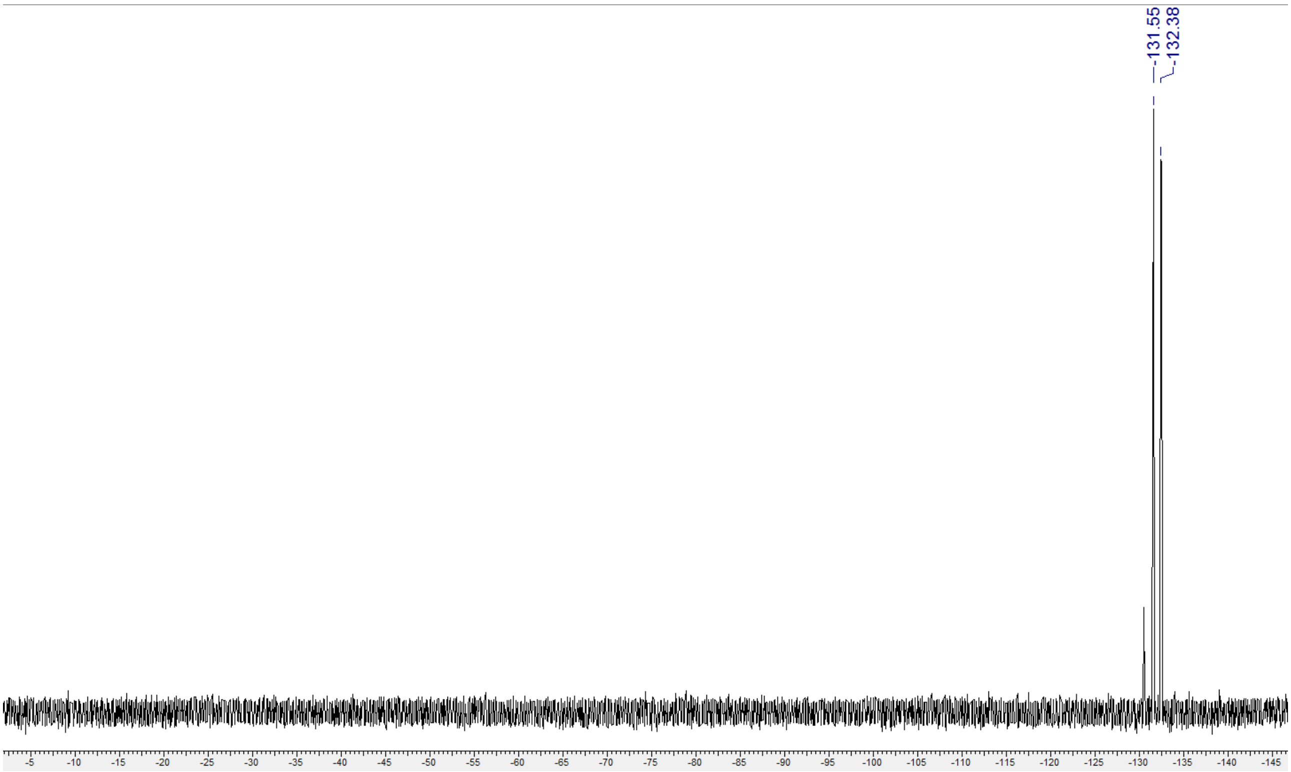

NMR ^{19}F spectrum (280 MHz, DMSO-D6) of compound **4f**.

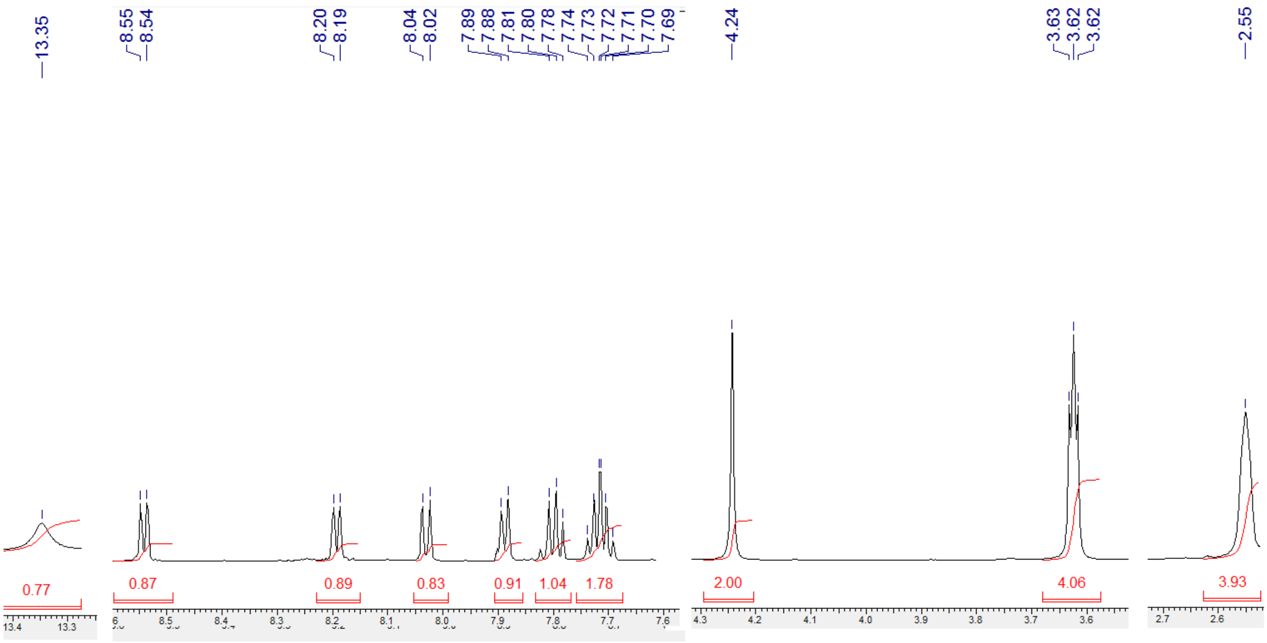

NMR ^{1}H spectrum (600 MHz, DMSO-D6) of compound **4m**.

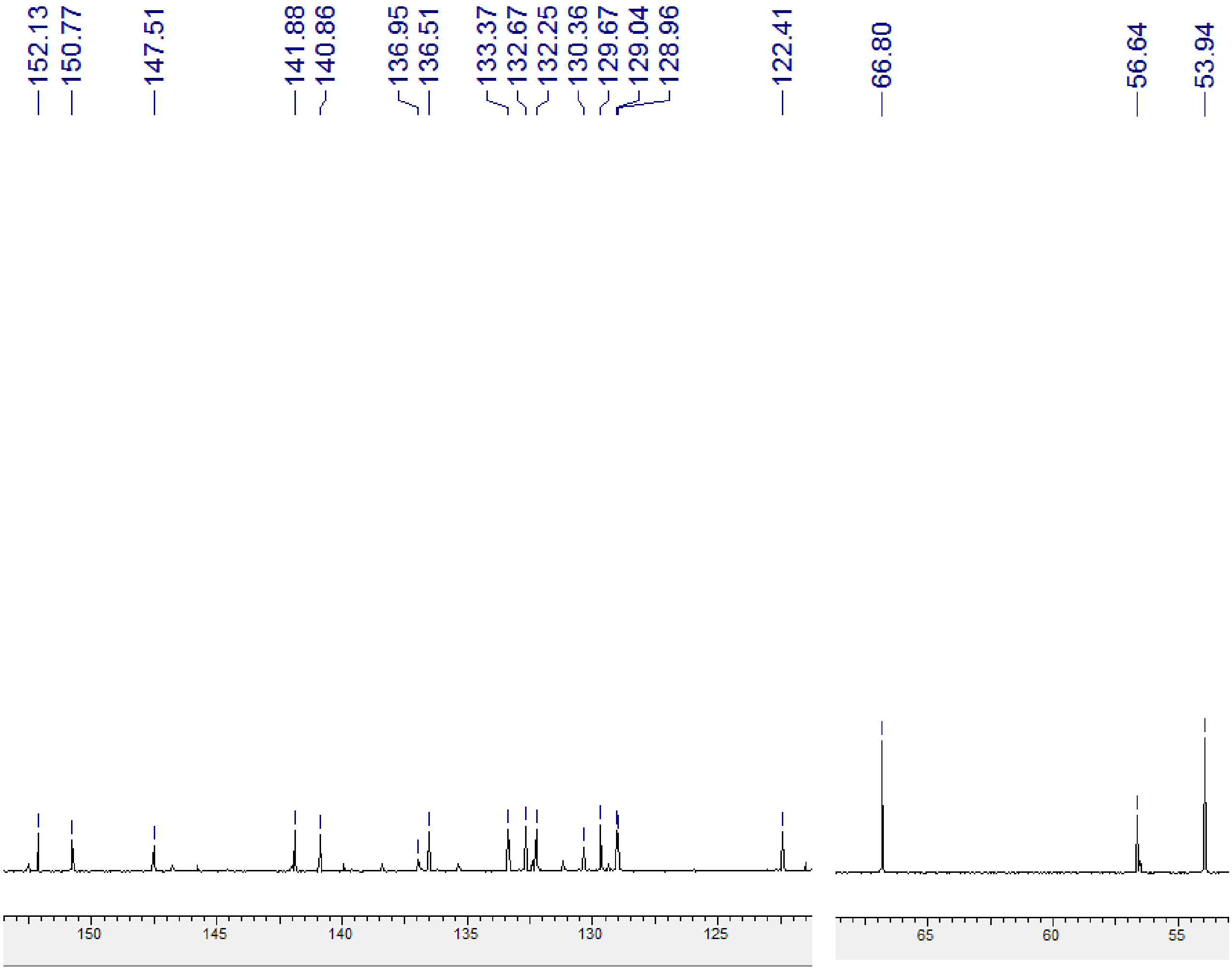

NMR ^{13}C spectrum (150 MHz, DMSO-D6) of compound **4m**.
